# Whole Transcriptome Analysis of Chicken Bursa Reveals Candidate Gene That Enhances the Host’s Immune Response to Coccidiosis

**DOI:** 10.3389/fphys.2020.573676

**Published:** 2020-10-30

**Authors:** Lijin Guo, Weiling Huang, Feng Tong, Xiaolan Chen, Sen Cao, Haiping Xu, Wei Luo, Zhenhui Li, Qinghua Nie

**Affiliations:** ^1^Department of Animal Genetics, Breeding and Reproduction, College of Animal Science, South China Agricultural University, Guangzhou, China; ^2^Guangdong Provincial Key Laboratory of Agro-Animal Genomics and Molecular Breeding, Key Laboratory of Chicken Genetics, Breeding and Reproduction, Ministry of Agriculture, Guangzhou, China; ^3^Guangdong Laboratory for Lingnan Modern Agricultural Science and Technology, South China Agricultural University, Guangzhou, China

**Keywords:** coccidiosis, RNA-seq, *TNFRSF6B*, macrophages polarization, macrophages apoptosis, Fas signal pathway, inflammatory response

## Abstract

Coccidiosis is a major hazard to the chicken industry, but the host’s immune response to coccidiosis remains unclear. Here, we performed *Eimeria* coccidia challenge in 28-day-old ROSS 308 broilers and selected the bursa from the three most severely affected individuals and three healthy individuals for RNA sequencing. We obtained 347 DEGs from RNA-seq and found that 7 upregulated DEGs were enriched in *Cytokine-cytokine receptor interaction* pathway. As the DEGs with the highest expression abundance in these 7 genes, *TNFRSF6B* was speculated to participate in the process of host’s immune response to coccidiosis. It is showed that *TNFRSF6B* can polarize macrophages to M1 subtype and promote inflammatory cytokines expression. In addition, the expression of *TNFRSF6B* suppressed HD11 cells apoptosis by downregulating Fas signal pathway. Besides, *TNFRSF6B*-mediated macrophages immunity activation can be reversed by apoptosis. Overall, our study indicates that *TNFRSF6B* upregulated in BAE, is capable of aggravating the inflammatory response by inhibiting macrophages apoptosis via downregulating Fas signal pathway, which may participate in host’s immune response to coccidiosis.

## Introduction

Chicken coccidiosis, a serious intracellular parasitic disease caused by *Eimeria*, is one of the most major livestock diseases (Morris and Gasser, 2006; Blake and Tomley, 2014). The poultry industry is considered as the most affected by coccidiosis and the annual cost of coccidiosis is estimated at more than 3 billion dollars (Dalloul and Lillehoj, 2006). The research of chickens breeding often focuses on the selection of the growth rate (Sheng et al., 2013) and appearance (Zheng et al., 2020), while less on the disease resistance. Compared to commercial breeds, local chicken breeds have the advantage of being more resistant to coccidia (Pinard-Van et al., 1998). The different Eimeria species invade different regions and this diversity leads to great challenges in controlling coccidiosis. Coccidia oocysts have a strong resistance to environmental, resulting that the use of drugs in actual production cannot eradicate the infection and only control the outbreak of the disease to a certain extent (Chapman and Jeffers, 2014). In addition, the *Eimeria* coccidia exhibit a complex life cycle (Quiroz-Castaneda and Dantan-Gonzalez, 2015), making it difficult for vaccines to be effective in the long term. The host’s immune response to coccidiosis mainly includes humoral immunity and cellular immunity (Min et al., 2013). Although both humoral immunity and cellular immunity can produce specific antibodies during coccidia infection, cellular immune response is the most critical for anti-coccidia infection (Yun et al., 2000).

The early findings indicate that T cells-mediated cellular immune responses play a crucial role in anticoccidial immunity (Wakelin et al., 1993; Min et al., 2013). The population of T cells can be induced by incremental interferon-γ (IFN-γ), which was considered as a kind of proinflammatory cytokine (Rose et al., 1991). In addition to producing IFN-γ, T cells also secrete a large number of secretions, such as the cytokine interleukin (IL), macrophage colony-stimulating factor (M-CSF), and tumor necrosis factor (TNF). The combination of IL-2 or IL-8 and vaccine can effectively protect against coccidia (Min et al., 2001; Lillehoj et al., 2005; Song et al., 2015). There was a positive correlation between the cecal lesion score and the expression level of IL-10 (Boulton et al., 2018). IL-10 has been reported to be associated with intestinal diseases (Kole and Maloy, 2014). Infection with *Eimeria maxima* could induce IL-10 expression in the spleen, and more importantly, it showed a higher expression level in susceptible chickens (Rothwell et al., 2004). IL-10 was the core factor for the pathogenesis of intercellular pathogens and intestine luminal IL-10 enabled *Eimeria* infection in broilers (Arendt et al., 2016). The chickens fed with the IL-10 antibody showed no obvious symptoms after challenge (Sand et al., 2016). IL-10 was widely believed to be involved in the development of coccidiosis (Arendt et al., 2016, 2019; Sand et al., 2016). These all highlight the importance of the cytokine’s cellular immune response in the host’s resistance to coccidia infection. Despite the increasing number of reports on coccidiosis, the chicken’s immune regulatory network against coccidiosis is still not comprehensive.

Tumor necrosis factor receptor super family 6 B (TNFRSF6B), also called decoy receptor (DCR3), is a kind of protein located on membrane surface without transmembrane structure (Pitti et al., 1998). TNFRSF6B is capable of binding to FasL and causes the regulation of Fas signal pathway, thus inhibiting the cells apoptosis (Li et al., 2007; Mabrouk et al., 2008). The ectopic expression of TNFRSF6B in tumor caused the inhibition of Fas-mediated cell apoptosis, resulting in the immune escape from immune process (Zhou et al., 2013; Zhang et al., 2015; Zhang X. et al., 2018). In addition, TNFRSF6B can regulate the proliferation of chondrocytes via ERK signaling and Fas-induced apoptosis (Hayashi et al., 2011). TNFRSF6B also acts regulatory role on inflammatory response in some intestinal diseases. It was showed that TNFRSF6B can enhance the intercellular interaction between macrophages and intestinal epithelial cells by upregulating CAM-1 (Philip et al., 2004). The aberrant expression of TNFRSF6B was confirmed a strong correlation with inflammatory bowel disease (Kugathasan et al., 2008; Funke et al., 2009; Henderson et al., 2011; Brant et al., 2017). These researches demonstrate a potential role of TNFRSF6B on avian intestinal diseases. However, there is still no any report of TNFRSF6B in chicken coccidiosis.

In this study, we carried out the Eimeria challenge in Ross 308 broilers and identified 347 DEGs through RNA sequencing. It was speculated that *TNFRSF6B* may participated in the immune response to chicken coccidiosis. We performed a series of experiments to verify its role, especially in macrophages immune activation and macrophages apoptosis. Totally, we confirmed that *TNFRSF6B* can aggravate the inflammatory response in chicken coccidiosis by inhibiting Fas signal pathway.

## Materials and Methods

### Ethics Statement

All animal experiments in our study were approved by the Animal Care Committee of Scotland’s Rural College (Project license number: 70–8213, 29 January, 2018). We carried out the animal experiments following the policies and rules formulated by the committee and in accordance with the Animal Protection Ordinance of Scotland’s Rural College.

### Samples Collection, RNA Extraction, Library Construction, and RNA-Seq

Twenty four Ross 308 commercial broilers were evenly distributed to six cages after birth and were fed *ad libitum*. At the age of 28-day, the feeding of chicken *Eimeria* coccidia oocysts (Intervet, Australia) was carried out on 12 broilers in three cages, which were considered to be the challenge treatment group (BAE), The dose of the *Eimeria* coccidia oocyst mixture is 1 mL for one individual. The 12 broilers in other three cages were treated with equal amount of saline, which were considered to be the control group (BAN). At day 35, all individuals were euthanized and intestinal lesion score (Johnson and Reid, 1970) was performed. The lesion phenotypic data and lesion score standard was listed in Supplementary Figure 1. Based on the lesion degree, the three individuals with most serious lesion in BAE and the three normal individuals were selected for mRNA sequencing. Bursa tissues were collected from the six individuals which has been selected, TRIzol Reagent (Life Technologies, Carlsbad, CA, United States) was used to extract total RNA by following manufacturer’s instruction. The integrity and concentration of RNAs were evaluated by NanoDrop 2100 (Thermo Fisher Scientific, Fremont, CA, United States). Then, NEBNext Poly (A) mRNA Magnetic Isolation Module (NEB, E7490) was used to isolate mRNA. The cDNA library was constructed following the instructions of NEBNext RNA Library Prep Kit for Illumina (NEB, E7530) and NEBNext Multiplex Oligos for Illumina (NEB, E7500). Subsequently, the mRNA was fragmented into the inserts of approximately 200 nt, for synthesizing the first-strand cDNA and the second cDNA. The double-stranded cDNA synthesis was performed with end-repair/dA-tail and adaptor ligation. The suitable fragments were isolated by Agencourt AMPure XP beads, and enriched by PCR. At last, the Illumina HiSeqTM sequencing platform was used for the sequencing of the constructed cDNA libraries. The raw data of RNA sequencing was submitted to National Center for Biotechnology Information database (accession ID: PRJNA561064; the accession link)^1^.

### Plasmid Construction and siRNA Synthesis

The complete CDS of *TNFRSF6B* was amplified by PCR and was cloned into pJET1.2/blunt clone vector (Thermo Fisher Scientific, United States). Then, the *TNFRSF6B* complete CDS was completely excised from the pJET1.2 plasmid by restriction enzyme and connected to the pcDNA3.1 overexpression vector by T4 DNA Ligase (Thermo Fisher Scientific, United States). The XhoI and XbaI sites were selected. The PCR forward primer sequence was “ATGTTCTTATATAACGCGCAGC” and the reverse was “CTACAAGAGGAAGCGCTCCCGT”. Small interfering RNA (siRNA; oligonucleotide sequence: GGGAGCGCTTCCTCTTGTA) of chicken *TNFRSF6B* gene was designed and synthesized (RiboBio, Guangzhou, China).

### Cell Culture

Chicken macrophage cells (HD11) were cultured with RPMI 1640 Medium (Gibco, United States) with 10% fetal bovine serum (Gibco, United States) and 0.2% penicillin/streptomycin (Invitrogen, United States) in a 37°C, 5% of CO_2_ incubator. Lipofectamine 3000 Reagent (Invitrogen, United States) was used in cell transfection following its protocol, when the cells grow to 70% confluence.

### Reverse Transcription and Quantitative PCR (qPCR) Analysis

Total RNA of cells was extracted following the chloroform method protocol. 1 mL MagZol Reagent (Magen, Guangzhou, China) was used for cell lysis. Then add 200 μL chloroform (YongDa Chemical, Tianjin, China) into the tube and shake 15 s, incubate in room temperature, centrifuge at 12,000 × *g* for 15 min at 4°C. After centrifugation, transfer the supernatant into another 1.5 mL tube, mix with 0.5 mL isopropanol (YongDa Chemical, Tianjin, China), incubate in room temperature for 10 min and centrifuge at 10,000 × *g* for 10 min at 4°C. Discard the supernatant, wash the sediment with 75% anhydrous ethanol (YongDa Chemical, Tianjin, China) and centrifuge at 7,500 × *g* for 5 min at 4°C. Subsequently, the supernatant was discarded and keep in the room temperature for 3 min. At last, 40 μL RNase-free water was added into the tube to dilute the RNA sediment and save at −80°C. The reserve transcription was performed following the protocol of HiScript III 1st Strand cDNA Synthesis Kit (Vazyme, Nanjing, China). The mRNA expression was detected in QuantStudio 5.0 (Thermo Fisher Scientific, United States) by using ChamQ Universal SYBR qPCR Master Mix (Vazyme, Nanjing, China). The internal control used in qPCR analysis is *GAPDH*. The information of primers used in qPCR analysis was listed in Supplementary Table 1. Three replicates were performed for qPCR analysis. 2^–ΔΔCT^ method was used to calculate the relative mRNA expression (ΔCT = CT_target gene_-CT_reference gene_, ΔΔCT = ΔCT_treated group_-ΔCT_negative control group_).

### Western Blot Assay

Cell protein extraction was performed by using ice-cold radio immunoprecipitation (RIPA) lysis buffer (Beyotime, Shanghai, China) with phenylmethylsulfonyl fluoride (PMSF) (Beyotime, Shanghai, China). The protein was separated in 12% SDS-PAGE and transferred to a polyvinylidene fluoride (PVDF) membrane (Bio-Rad, United States). Then, the membrane was incubated with QuickBlock^TM^ Blocking Buffer for Western Blot (Beyotime, Shanghai, China). After that, the target protein was probed using antibodies. The information of antibodies used in this study was follow: the dilution of Cleaved-Caspase 8/p43/p18 Antibody (Proteintech, United States) is 1:500, the dilution of Rabbit Anti-caspase 9/p10 antibody (Bioss, Shanghai, China) is 1:500, the dilution of Rabbit Anti-PARP 1 antibody (Bioss, Shanghai, China) is 1:500, the dilution of Rabbit Anti-Lamin B antibody (Bioss, Shanghai, China) is 1:500 and Mouse anti-GAPDH Antibody (Boster, Wuhan, China) is 1: 2,000. Subsequently, the secondary antibodies with HRP anti-rabbit/mouse IgG antibody (Boster, Wuhan, China) was used for incubation in a 1:10,000 dilution. Finally, DAB Horseradish Peroxidase Color Development Kit (Beyotime, Shanghai, China) was used for color rendering in the Odyssey Fc Image System (LI-COR, United States).

### Flow Cytometric Analysis for Cell Apoptosis

Cells was collected after the digestion by 0.25% trypsin (Gibco, United States). Then, the cells were stained by using an Annexin V-FITC apoptosis detection kit (Beyotime, Shanghai, China) following its protocol. Finally, the cells were analyzed through the flow cytometer (Beckman, United States) following the standard process.

### ELISA Analysis Assay

ELISA Kits (MLbio, Shanghai, China) were used to measure the cytokines secretion level following the protocol. The cells supernatant was collected in a 1.5 mL tube. After the centrifugation, transfer the supernatant into a new 1.5 mL tube. The supernatant and biotin-label antibodies were incubated in the same time. After washing, add the HRP marked with avidin. Then, color rendering was performed. Finally, absorption was measured in a Microplate Reader (BioTek, United States).

### Reactive Oxygen Species (ROS) Assay

Reactive Oxygen Species Assay Kit (Beyotime, Shanghai, China) was used to detect the ROS level by following instruction protocol. The cells were washed by PBS and incubated with DCFH-DA at 37°C for 30 min. Then, the cells were further washed three times by PBS. DMi8 Fluorescence Microscope (Leica, Germany) was used to capture the random interfaces to visualize the ROS level. In addition, Microplate Reader (BioTek, United States) was used to measure the fluorescence value at 480/528 nm.

### Nitric Oxide (NO) Assay

Nitric Oxide Assay Kit (Beyotime, Shanghai, China) was used to measure the level of NO by following manufacture’s protocol. The cell supernatant sample were collected into a 1.5 mL centrifuge tube and centrifuged at 12,000 × *g* for 5 min. Then, we transferred the supernatant into another new 1.5 mL tube and discarded the sediment. The samples and reference samples were put into the 96-well plate in 50 μL volume. Subsequently, we added 50 μL Griess Reagent I and 50 μL Griess Reagent II into the wells. Finally, absorbance was measured in a Microplate Reader (BioTek, United States) at 540 nm.

### Statistical Analysis

All data was showed as mean ± standard deviation (S.E.M.). There were three replicates in every group of all experiment. Statistical significance of the mean difference was assessed using unpaired two-sample *t*-tests. ^∗^*P* < 0.05; ^∗∗^*P* < 0.01; NC, negative control.

## Results

### Summary of Transcriptome Data and Differentially Expressed Genes (DEGs) Validation

**FIGURE 1:**
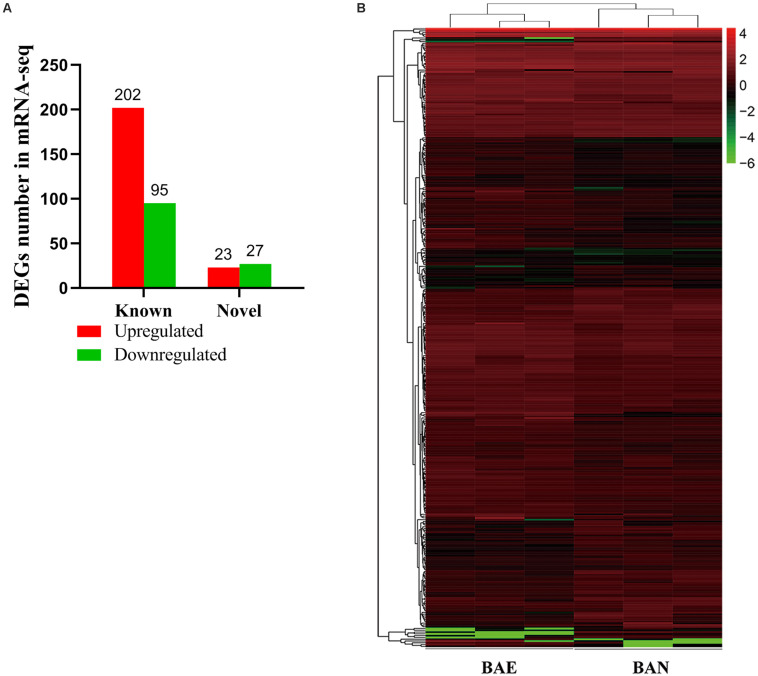
Blast Analysis of Transcriptome from RNA-seq. **(A)** The quantity statistics of DEGs, in which the red column representing upregulated DEGs and the green representing downregulated DEGs. **(B)** Heatmap of 347 differentially expressed genes (DEGs) between BAE and BAN, in which rows representing genes and columns representing different groups.

According to the lesion degree (Supplementary Figure 1), bursa total RNA was extracted from three individuals with the most severe lesions and three healthy individuals for RNA-seq. All raw data was submitted to NCBI database (accession ID: PRJNA561064). In this mRNA sequencing, we obtained at least 62.56 million clean reads for each sample, which has passed the filter. As shown in Supplementary Table 2, the pair-end reads were no less than 31.28 million, the number of clean bases was no less than 9.34 billion. The GC content ranged from 52.17 to 52.65%. The percentage of bases whose base recognition accuracy reaches Q30 standard (the accuracy of base recognition ≥99.9%) is not less than 94.51%. The clean reads were mapped to chicken GRCg6a genome, the multiple mapped reads rate ranged from 3.90 to 4.13% and unique mapped rate ranged from 88.62 to 90.05% (Supplementary Table 3). A total of 19,866 genes were detected, including 16,231 known genes and 3,635 novel genes (Supplementary Table 4). In this study, | (Fold change) | >1.5 and *P*-value < 0.05 were set as the cut-off standard for differentially expressed genes and 347 DEGs were identified, including 297 known genes and 50 novel genes. Among these DEGs, 225 genes were upregulated and 122 genes were downregulated in ROSS 308 broiler bursa after challenge with *Eimeria* oocysts (Figure 1A). All DEGs were listed in Supplementary Table 5.

Among of all DEGs, the most highly expressed gene was cathelicidin-B1-like (*CATHB1*), and it was upregulated in BAE group (log_2_FC = 0.82, *P*-value = 2.66E^–5^), which may be associated with intestinal immune system. Moreover, *CATHB1* was also the most highly expressed gene in chicken bursa of fabricius under the stress of corticosterone hormone (Zhang Y. et al., 2018). In BAE group, the top 5 highly expressed known DEGs included *CATHB1*, retinoic acid receptor responder 1 (*RARRES1*), transferrin (*TF*), extracellular fatty acid-binding protein (*LCN8*) and keratin 24 (*KRT24*). In all of the downregulated known DEGs, pre-mRNA-processing factor 19-like (*LOC107053341*) was the most highly expressed gene (log_2_FC = −2.00, *P*-value = 8.99E^–5^), as well the unique down-regulated DEG among the top 10 known DEGs. The cluster analysis of DEGs in heat map showed that the gene expression pattern was similar intra-group, while were different between groups (Figure 1B).

**FIGURE 2:**
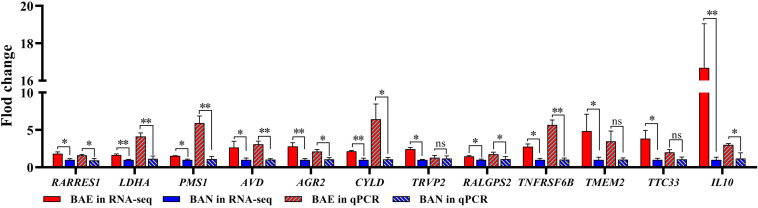
qPCR Validation for DEGs from RNA-seq. 12 DEGs were randomly selected for qPCR validation. The data was shown as mean ± SEM; *P < 0.05, **P < 0.01, ns: no significant difference.

In order to confirm the DEGs from RNA-seq, 12 DEGs were randomly chose for qPCR validation. The RNA used in qPCR experiments was from the same samples for RNA-seq. The 12 selected DEGs included *RARRES1*, *LDHA*, *PMS1*, *AVD*, *AGR2*, *CYLD*, *TRVP2*, *RALGPS2*, *TNFRSF6B*, *TMEM2*, *TTC33*, and *IL10*. qPCR validation results showed that these DEGs relative expression levels were consistent with those in RNA-seq (Figure 2).

### GO and KEGG Pathway Analysis for DEGs

**FIGURE 3:**
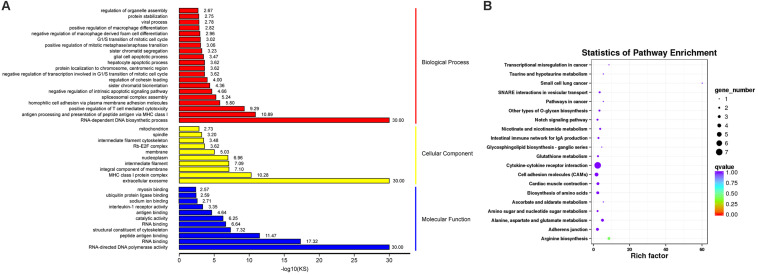
GO and KEGG Analysis for DEGs. **(A)** The top GO terms for DEGs, in which rows representing GO terms and columns representing the value of “–log10(KS)”. **(B)** The top 20 enriched pathways in KEGG pathway analysis for DEGs, in which rows representing pathways and columns representing the percentage (%) of rich factor.

Gene ontology (GO) functional enrichment of DEGs was performed. According to functional annotation, they were divided into three sections, including *Biological Process* (BP), *Cellular Component* (CC), and *Molecular Function* (MF). Kolmogorov-Smirnov (KS) value <0.05 was considered as the criterion for significant enrichment. The DEGs could be classified in 1776 terms for BP, 234 terms for CC and 356 terms for MF. Among of them, 95 terms, 26 terms, and 63 terms were significantly enriched, respectively (Supplementary Table 6). Some terms that might be associated with coccidiosis, were enriched, including *antigen processing and presentation of peptide antigen via MHC class I*, *positive regulation of T cell mediated cytotoxicity*, *negative regulation of macrophage derived foam cell differentiation*, *positive regulation of macrophage differentiation*, *MHC class I protein complex* and *interleukine-1 receptor activity* (Figure 3A).

In addition, KEGG pathway analysis was carried out. The DEGs were observed to be enriched in 88 pathways. The *q*-value ≤ 1 was regarded as a significant difference in statistics of pathway enrichment. The top 20 enriched pathways were shown in Figure 3B and listed in Table 1. It was showed that these DEGs were enriched in *Cytokine-cytokine receptor interaction* and *Intestinal immune network for IgA production*, of which might be involved in chicken coccidiosis. Based on the situation of dominance of T-cells mediated immunity in anticoccidial response, we pay more attention to the DEGs enriched in *Cytokine-cytokine receptor interaction* pathway. We obtained a total of 7 DEGs enriched in this pathway, including *TNFRSF6B*, *IL10*, *TNFRSF25*, *IL12RB2*, *IL1R1*, *TNFSF13B*, and *AMHR2* (Table 2). It is noteworthy that these 7 DEGs were all upregulated. *IL10* has been widely studied in chicken coccidiosis and its upregulation in serum was regarded as a symbol of intestinal inflammatory damage caused by *Eimeria* coccidia (Sand et al., 2016). In our mRNA sequencing, *IL10* indeed was regulated. However, among the 7 DEGs, *TNFRSF6B* showed the highest abundance and its specific role in coccidiosis has not been reported. Whether it plays a role in coccidiosis-mediated intestinal inflammatory injury is unclear. As a member of *Cytokine-cytokine receptor interaction* pathway, also a kind of protein located in the surface of cell membrane, it was speculated that *TNFRSF6B* gene may participate in the immune response in coccidiosis and we assumed it was a potential candidate for subsequent research.

**TABLE 1:** The top 20 enriched KEGG pathways.

**Pathway**	**Enrichment factor**	***Q* value**	**Gene name**
Arginine biosynthesis	8.59	0.42	*GLUL, ASS1, ASL*
Small cell lung cancer	60.11	1.00	*MAX*
Alanine, aspartate and glutamate metabolism	5.01	1.00	*ASS1, ASL, GLUL*
Cytokine-cytokine receptor interaction	2.36	1.00	*IL12R2, IL10, TNFRSF25, TNFRSF6B, TNFSF13B, AMHR2, IL1R1*
Nicotinate and nicotinamide metabolism	3.76	1.00	*NT5E, NADK*
Transcriptional misregulation in cancer	8.59	1.00	*MAX*
SNARE interactions in vesicular transport	3.44	1.00	*STX5, STX3*
Cardiac muscle contraction	2.50	1.00	*ATP1B, Gallus_gallus_newGene_1, Gallus_gallus_newGene_5066*
Other types of O-glycan biosynthesis	3.34	1.00	*FNG, POFUT*
Biosynthesis of amino acids	2.40	1.00	*ASS1, ASL, GLUL*
Adherens junction	2.23	1.00	*SRC, TGFBR2, PVRL4*
Intestinal immune network for IgA production	2.80	1.00	*IL-10, TNFSF13B*
Pathways in cancer	5.46	1.00	*MAX*
Taurine and hypotaurine metabolism	5.46	1.00	*GGT1*
Ascorbate and aldarate metabolism	5.46	1.00	*UGT*
Glutathione metabolism	2.50	1.00	*GGT1, GPX*
Glycosphingolipid biosynthesis – ganglio series	4.62	1.00	*SIAT9*
Cell adhesion molecules (CAMs)	1.73	1.00	*PDCD1, CLDN, SELPLG, SELE*
Amino sugar and nucleotide sugar metabolism	2.31	1.00	*NANP, CHIA*
Notch signaling pathway	2.27	1.00	*FNG, CTBP*

**TABLE 2:** DEGs enriched in *Cytokine-cytokine receptor interaction* pathway.

**Gene name**	**Gene description**	**BAE FPKM**	**BAN FPKM**	***P*-value**	**log2FC**	**Regulated**
*TNFRSF6B*	TNF receptor superfamily member 6b	14.22	5.13	0.00	1.41	Up
*AMHR2*	Anti-mullerian hormone receptor type 2	5.52	1.90	0.02	1.29	Up
*IL10*	Interleukin 10	4.87	0.29	0.00	3.76	Up
*TNFSF13B*	Tumor necrosis factor superfamily member 13b	3.85	1.44	0.00	1.40	Up
*TNFRSF25*	TNF receptor superfamily member 25	3.20	1.66	0.03	0.98	Up
*IL1R1*	Interleukin 1 receptor type 1	0.97	0.37	0.03	1.37	Up
*IL12RB2*	Interleukin 12 receptor subunit beta 2	0.89	0.56	0.00	1.43	Up

### TNFRSF6B Polarizes Macrophages to M1 Subtype

**FIGURE 4:**
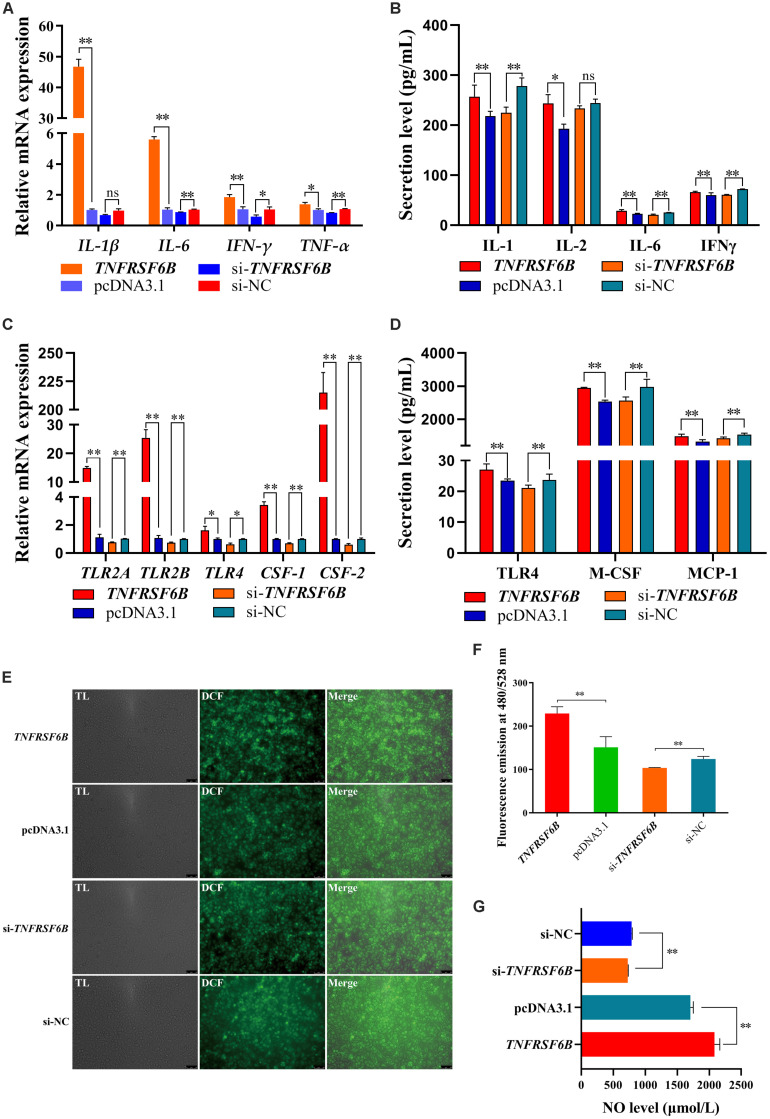
*TNFRSF6B* polarizes macrophages to M1 subtype. **(A)** The mRNA expression of M1 macrophages polarization marker genes was upregulated or downregulated after the overexpression or knockdown of *TNFRSF6B* by qPCR. **(B)** The secretion of IL-1, IL-2, IL-6, and IFNγ from HD11 cells after the transfection with pcDNA3.1-*TNFRSF6B* and si-*TNFRSF6B* by ELISA analysis. **(C)** The mRNA expression of macrophages activation-related genes was upregulated or downregulated after the overexpression or knockdown of *TNFRSF6B* by qPCR. **(D)** The secretion of TLR4, M-CSF, and MCP-1 in cell supernatant after the transfection with pcDNA3.1-*TNFRSF6B* and si-*TNFRSF6B* by ELISA analysis. **(E)** ROS level analysis in HD11 cells with the overexpression of *TNFRSF6B*. **(F)** ROS level analysis in HD11 cells with the knockdown of *TNFRSF6B*. **(G)** NO level detection after the overexpression or knockdown of *TNFRSF6B*. The data was shown as mean ± SEM; *P < 0.05, **P < 0.01, ns, no significant difference.

To verify the role of *TNFRSF6B* on cell level, we constructed an overexpress plasmid and synthesized its siRNA. The innate immune system responds to parasites by proinflammatory cytokines, in which process macrophages polarization plays a critical role (Dobbs et al., 2020). Thus, we investigated the effect of *TNFRSF6B* on macrophages polarization. After the 48-h transfection in HD11 cells with pcDNA3.1-*TNFRSF6B* and si-*TNFRSF6B*, we quantified the relative mRNA expression of some genes related to M1 subtype polarization. It is showed that the overexpression of *TNFRSF6B* upregulates the mRNA expression of *IL-1*β, *IL-2*, *IL-6*, *IFN-*γ, and *TNF-*α (Figure 4A). Similarly, ELISA assay was performed and the results showed that *TNFRSF6B* could promote the secretion of IL-1, IL-6, and IFN-γ (Figure 4B). The upregulation of these markers indicates a positive role of *TNFRSF6B* in polarizing macrophages to M1 subtype. The results further demonstrate that *TNFRSF6B* may play a positive role in the promotion of M1 macrophage polarization.

Based on the situation of *TNFRSF6B* positive role on M1 macrophage polarization, we detected the relative mRNA expression of toll like receptor 2 A (*TLR2A*), toll like receptor 2 B (*TLR2B*), toll like receptor 4 (*TLR4*), colony-stimulating factor-1 (*CSF-1*) and colony-stimulating factor-2 (*CSF-2*). It is showed that *TNFRSF6B* expression upregulates the mRNA level of *TLR2A*, *TLR2B*, *TLR4*, *CSF-1* and *CSF-2* by leaps and bounds (Figure 4C). Furthermore, *TNFRSF6B* increased observably the secretion of TLR4, M-CSF and MCP-1 (Figure 4D). Besides, Reactive Oxygen Species level and NO level were increased significantly by *TNFRSF6B* (Figures 4E–G).

### The Apoptosis Reversed the TNFRSF6B-Mediated Strengthening Effect on the Immune Response of Macrophages

**FIGURE 5:**
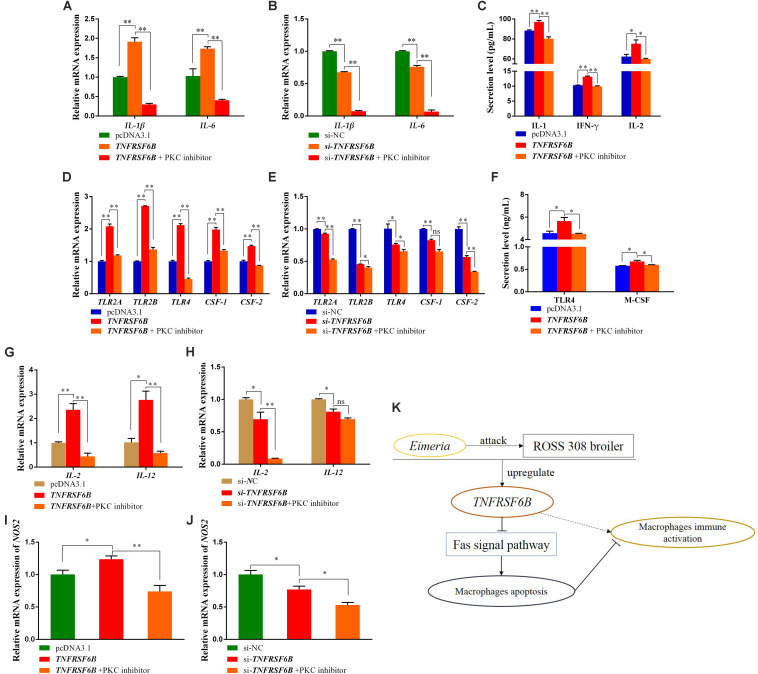
The Apoptosis reversed the TNFRSF6B-mediated strengthening effect on the immune response of macrophages. **(A)** The mRNA expression of *IL-1*β and *IL-6* after the treatment of *TNFRSF6B* overexpression and PKC inhibitor. **(B)** The mRNA expression of *IL-1*β and *IL-6* after the treatment of *TNFRSF6B* knockdown and PKC inhibitor. **(C)** The secretion levels of IL-1, IFN-γ, and IL-2 after the treatment of *TNFRSF6B* overexpression and PKC inhibitor. **(D)** The mRNA expression of macrophages activation-related genes after the treatment of *TNFRSF6B* overexpression and PKC inhibitor. **(E)** The mRNA expression of macrophages activation-related genes after the treatment of *TNFRSF6B* knockdown and PKC inhibitor. **(F)** The secretion levels of TLR4 and M-CSF after the treatment of *TNFRSF6B* overexpression and PKC inhibitor. **(G)** The mRNA expression of *IL-2* and *IL-12* after the treatment of *TNFRSF6B* overexpression and PKC inhibitor. **(H)** The mRNA expression of *IL-2* and *IL-12* after the treatment of *TNFRSF6B* knockdown and PKC inhibitor. **(I)** The mRNA expression of *NOS2* after the treatment of *TNFRSF6B* overexpression and PKC inhibitor. **(J)** The mRNA expression of *NOS2* after the treatment of *TNFRSF6B* knockdown and PKC inhibitor. **(K)** Model of *TNFRSF6B*-mediated macrophages immune activation. The data was shown as mean ± SEM; *P < 0.05, **P < 0.01, ns, no significant difference.

It was showed that *TNFRSF6B* was capable of antagonizing macrophage apoptosis by inhibiting Fas signal pathway (Supplementary Data 1). Base on the situation of M1 macrophages activation and apoptosis inhibition caused by *TNFRSF6B*, we further explored the relationship between them. We used PKC inhibitor to induce HD11 cell apoptosis to antagonize its anti-apoptosis caused by the downregulation of Fas signal pathway. It could be found that the mRNA expression of *IL-1*β and *IL-6* was declined by the treatment with PKC inhibitor (Figures 5A,B). The secretion of IL-1 and IFN-γ from HD11 cells was also downregulated (Figure 5C). The *TNFRSF6B*-mediated upregulation of these markers reflects the role of *TNFRSF6B* on polarizing macrophages to M1 subtype, but this process can be restored by apoptosis.

Again, it was found that the *TNFRSF6B*-mediated upregulation of *TLR2A*, *TLR2B*, *TLR4*, *CSF-1*, and *CSF-2* was restored or reversed by apoptosis (Figures 5D,E). The secretion of TLR4 and M-CSF, which had been promoted by *TNFRSF6B*, fell down to normal level (Figure 5F). When the apoptosis inhibition caused by *TNFRSF6B* was receded, the effect of *TNFRSF6B* on macrophages immune activation was limited. The mRNA level of *IL-2* and *IL-12* was also declined dramatically (Figures 5G,H). In addition, *NOS2* mRNA expression also showed similar changes (Figures 5I,J). The results suggest that *TNFRSF6B* mediates macrophages activation by inhibiting macrophages apoptosis.

## Discussion

In recent years, there have been increasing reports on the crucial role of cytokines in chicken coccidiosis infection (Del et al., 2014; Song et al., 2015; Jing et al., 2019), also in toxoplasma diseases (Lima et al., 2018; Pandori et al., 2019). Chicken IFN-γ and IL-2 were able to effectively improve the DNA vaccines against to coccidiosis (Song et al., 2015). Chicken IL-17 played a proinflammatory role in coccidiosis (Min et al., 2013). In this study, we performed the challenge with *Eimeria* coccidia in 28-day-old ROSS 308 broilers. In mRNA sequencing of bursa, we obtained 347 DEGs, of which 225 DEGs were upregulated, and a total of 7 DEGs were enriched in *Cytokine-cytokine receptor interaction* pathway. Among of the 7 DEGs, *IL-10* is widely believed to be involved in the development of coccidiosis (Arendt et al., 2016, 2019; Sand et al., 2016). In addition, *TNFRSF6B* showed the highest expression abundance in the 7 DEGs. Nevertheless, there is no literature about *TNFRSF6B* in coccidiosis.

*TNFRSF6B* was regarded as a pivotal molecule in inflammatory reaction and *TNFRSF6B* was pleiotropic in the inflammation regulation (Lin and Hsieh, 2011; Hsieh and Lin, 2017). *TNFRSF6B* was also considered as a kind of immunomodulator (Rothwell et al., 2004; Hsu et al., 2005; Liu et al., 2017). As a member of cytokine-cytokine receptor interaction pathway, *TNFRSF6B* also has an important role in various immune regulatory processes. Aberrant expression of *TNFRSF6B* in cancer cells inhibited Fas-mediated and TNFRSF14-mediated apoptosis, leading the cancer cells to escape during the immune process (Zhang et al., 2015; Zhang X. et al., 2018). *TNFRSF6B* was reported to associate with cell adhesion by increasing CAM-1 (Philip et al., 2004; Tsai et al., 2018). In macrophages, ICAM-1 expression is mediated by activation of NF-κB signaling (Yang et al., 2005), which promotes inflammatory response. In our research, the challenge with *Eimeria* coccidia upregulated the expression of *TNFRSF6B* and the upregulation of *TNFRSF6B* mediated the TLRs expression. TLRs play an important role in the identification of pathogens by immune cells. The accumulation of TLRs on the surface of macrophage membranes can promote cell immune activation and mediate cell inflammation (Fujiwara and Kobayashi, 2005). Furthermore, MCP-1 was also upregulated by the upregulation of *TNFRSF6B.* MCP-1 can promote the activation of macrophages, which results in high expression of TLRs on the surface of the macrophages’ cell membrane (Fujiwara and Kobayashi, 2005; Yadav et al., 2006; Fujiwara et al., 2019). Coccidioides infection activates the chicken’s immune system, leading to inflammatory damage in the gut (Shirley et al., 2005). This inflammatory response is primarily mediated by cytokines (Yun et al., 2000; Min et al., 2013). The expression and secretion of multiple pro-inflammatory cytokines caused by *TNFRSF6B* upregulation may mediate inflammation in coccidiosis.

TNFRSF6B has been demonstrated to drive M2-type differentiation in macrophages in vitro studies (Lin and Hsieh, 2011). In DcR3-treated macrophages, proinflammatory cytokines in response to lipopolysaccharide is impaired (Chang et al., 2004). DcR3 was also characterized with the function of activating microglia into an anti-inflammatory M2 phenotype in Alzheimer’s disease (Liu et al., 2017). However, *TNFRSF6B* showed a proinflammatory effect in chicken macrophages in our study. It may be due to species differences or large differences in homology. After *Eimeria* challenge, the proinflammatory cytokine expression would increase, also NO, which is involved in inflammatory response (Laurent et al., 2001). Proinflammatory cytokine secretion could be increased by *TNFRSF6B* in a co-stimulatory way (Lee et al., 2008). In our study, *TNFRSF6B* promoted various proinflammatory cytokines expression and NO secretion, which may be the cause of intestinal inflammation injury caused by coccidiosis. Not only does the inflammatory response, but the state of oxidative stress in the blood also plays a role in the physiopathology of coccidiosis (Griss et al., 2019). Serum level of ROS was higher than normal level after coccidia infection (Griss et al., 2019). The *TNFRSF6B*-mediated upregulation of ROS level may also be another way of intestinal damage in coccidiosis.

TNFRSF6B-mediated inhibition of apoptosis lead to overactivation of T cells, causing an autoinflammatory response (Lee et al., 2008). It is widely reported that TNFRSF6B can competitively bind to FasL with Fas and thus suppresses Fas signal-mediated apoptosis (Li et al., 2007; Mabrouk et al., 2008). Our study showed that the overexpression of *TNFRSF6B* observably suppresses macrophages apoptosis via antagonism on Fas signal pathway. In the recovery verification, we found that there was a negative correlation between macrophages apoptosis and *TNFRSF6B*-mediated proinflammatory effect. This finding is the first to confirm that *TNFRSF6B*-mediated proinflammatory effect is caused by the inhibition of macrophages apoptosis. In systemic lupus erythematosus, elevated TNFRSF6B may be associated with enhanced T cell activation and plays a possible role by T cell hyperreactivity and apoptosis inhibition in activated T cells (Lee et al., 2008), which is similar to our findings. Specific regulatory mechanisms may require further research.

## Conclusion

We found that *TNFRSF6B* is upregulated after coccidia challenge, and it can reduce macrophages apoptosis by down-regulating the Fas signaling pathway and thereby promoting macrophage M1 type polarization and aggravating the inflammatory response (Figure 5K).

## Data Availability Statement

The datasets presented in this study can be found in online repositories. The names of the repository/repositories and accession number(s) can be found below: https://www.ncbi.nlm.nih.gov/, PRJNA561064.

## Ethics Statement

The animal study was reviewed and approved by Animal Care Committee of Scotland’s Rural College. Written informed consent was obtained from the owners for the participation of their animals in this study.

## Author Contributions

LG carried out almost all experiments, participated in research design, and drafted the manuscript. WH was responsible for part of the experiments and drafted the part of the manuscript. FT participated in the data analysis and discussion of the results. XC was responsible for RNA-seq analysis. SC participated in the samples collection. WL and HX participated in analyzing the data. ZL participated in some verification experiments and manuscript drafting. QN designed the whole study and ensured the progress of the research. All authors contributed to the article and approved the submitted version.

## Conflict of Interest

The authors declare that the research was conducted in the absence of any commercial or financial relationships that could be construed as a potential conflict of interest.

## Footnotes

1 https://www.ncbi.nlm.nih.gov/bioproject/PRJNA561064

## Supplementary Material

Supplementary Figure 1 The lesion phenotypic and lesion score standard.

Supplementary Figure 2 *TNFRSF6B* inhibits macrophages apoptosis by downregulating Fas signal pathway.

Supplementary Table 1 Primers used for qPCR.

Supplementary Table 2 Data statistics.

Supplementary Table 3 Clean reads mapped efficiency.

Supplementary Table 4 Gene count statistics in RNA sequencing.

Supplementary Table 5 DEGs between BAE and BAN.

Supplementary Table 6 GO enrichment for DEGs.

Supplementary Data 1 *TNFRSF6B* inhibits macrophages apoptosis by downregulating Fas signal pathway.

## References

[B1] Arendt M. Elissa J. Schmidt N. Michael E. Potter N. Cook M. (2019). Investigating the role of interleukin 10 on *Eimeria* intestinal pathogenesis in broiler chickens. *Vet. Immunol. Immunopathol.* 218:109934. 10.1016/j.vetimm.2019.109934 31520870 PMC6861699

[B2] Arendt M. K. Sand J. M. Marcone T. M. Cook M. E. (2016). Interleukin-10 neutralizing antibody for detection of intestinal luminal levels and as a dietary additive in *Eimeria* challenged broiler chicks. *Poult. Sci.* 95 430–438. 10.3382/ps/pev365 26839414 PMC6592417

[B3] Blake D. P. Tomley F. M. (2014). Securing poultry production from the ever-present *Eimeria* challenge. *Trends Parasitol.* 30 12–19. 10.1016/j.pt.2013.10.003 24238797

[B4] Boulton K. Nolan M. J. Wu Z. Psifidi A. Riggio V. Harman K. (2018). Phenotypic and genetic variation in the response of chickens to *Eimeria tenella* induced coccidiosis. *Genet. Sel. Evol.* 50:63. 10.1186/s12711-018-0433-7 30463512 PMC6249784

[B5] Brant S. R. Okou D. T. Simpson C. L. Cutler D. J. Haritunians T. Bradfield J. P. (2017). Genome-wide association study identifies african-specific susceptibility loci in African Americans with inflammatory bowel disease. *Gastroenterology* 152 206–217. 10.1053/j.gastro.2016.09.032 27693347 PMC5164948

[B6] Chang Y. C. Hsu T. L. Lin H. H. Chio C. C. Chiu A. W. Chen N. J. (2004). Modulation of macrophage differentiation and activation by decoy receptor 3. *J. Leukoc. Biol.* 75 486–494. 10.1189/jlb.0903448 14657214

[B7] Chapman H. D. Jeffers T. K. (2014). Vaccination of chickens against coccidiosis ameliorates drug resistance in commercial poultry production. *Int. J. Parasitol. Drugs Drug Resist.* 4 214–217. 10.1016/j.ijpddr.2014.10.002 25516830 PMC4266793

[B8] Dalloul R. A. Lillehoj H. S. (2006). Poultry coccidiosis: recent advancements in control measures and vaccine development. *Expert Rev. Vac.* 5 143–163. 10.1586/14760584.5.1.143 16451116

[B9] Del C. E. Gallego M. Lillehoj H. S. Quilez J. Lillehoj E. P. Ramo A. (2014). IL-17A regulates *Eimeria tenella* schizont maturation and migration in avian coccidiosis. *Vet. Res.* 45:25. 10.1186/1297-9716-45-25 24571471 PMC3975951

[B10] Dobbs K. R. Crabtree J. N. Dent A. E. (2020). Innate immunity to malaria-the role of monocytes. *Immunol. Rev.* 293 8–24. 10.1111/imr.12830 31840836 PMC6986449

[B11] Fujiwara M. Matoba T. Koga J. I. Okahara A. Funamoto D. Nakano K. (2019). Nanoparticle incorporating Toll-like receptor 4 inhibitor attenuates myocardial ischaemia-reperfusion injury by inhibiting monocyte-mediated inflammation in mice. *Cardiovasc. Res.* 115 1244–1255. 10.1093/cvr/cvz066 30851101

[B12] Fujiwara N. Kobayashi K. (2005). Macrophages in inflammation. *Curr. Drug Targets Inflamm. Allergy* 4 281–286. 10.2174/1568010054022024 16101534

[B13] Funke B. Autschbach F. Kim S. Lasitschka F. Strauch U. Rogler G. (2009). Functional characterisation of decoy receptor 3 in Crohn’s disease. *Gut* 58 483–491. 10.1136/gut.2008.148908 19039087

[B14] Griss L. G. Galli G. M. Fracasso M. Silva A. D. Fortuoso B. Schetinger M. (2019). Oxidative stress linked to changes of cholinesterase and adenosine deaminase activities in experimentally infected chicken chicks with *Eimeria* spp. *Parasitol. Int.* 71 11–17. 10.1016/j.parint.2019.03.003 30849474

[B15] Hayashi S. Nishiyama T. Miura Y. Fujishiro T. Kanzaki N. Hashimoto S. (2011). DcR3 induces cell proliferation through MAPK signaling in chondrocytes of osteoarthritis. *Osteoarthr. Cartil.* 19 903–910. 10.1016/j.joca.2011.03.005 21420502

[B16] Henderson P. van Limbergen J. E. Wilson D. C. Satsangi J. Russell R. K. (2011). Genetics of childhood-onset inflammatory bowel disease. *Inflamm. Bowel Dis.* 17 346–361. 10.1002/ibd.21283 20839313

[B17] Hsieh S. L. Lin W. W. (2017). Decoy receptor 3: an endogenous immunomodulator in cancer growth and inflammatory reactions. *J. Biomed. Sci.* 24:39. 10.1186/s12929-017-0347-7 28629361 PMC5477258

[B18] Hsu T. L. Wu Y. Y. Chang Y. C. Yang C. Y. Lai M. Z. Su W. B. (2005). Attenuation of Th1 response in decoy receptor 3 transgenic mice. *J. Immunol.* 175 5135–5145. 10.4049/jimmunol.175.8.5135 16210617

[B19] Jing L. Yu Z. Gao X. Liu C. Lv X. Zheng S. (2019). Inhibition of tumor necrosis factor alpha and increased of interleukin 10 by *Lactobacillus*: a molecular mechanism protection against TNBS-induced ulcerative colitis in chicks. *Immunopharmacol. Immunotoxicol.* 41 1–6. 10.1080/08923973.2019.1566360 30821556

[B20] Johnson J. Reid W. M. (1970). Anticoccidial drugs: lesion scoring techniques in battery and floor-pen experiments with chickens. *Exp. Parasitol.* 28 30–36. 10.1016/0014-4894(70)90063-9 5459870

[B21] Kole A. Maloy K. J. (2014). Control of intestinal inflammation by interleukin-10. *Curr. Top. Microbiol. Immunol.* 380 19–38. 10.1007/978-3-662-43492-5_2 25004812

[B22] Kugathasan S. Baldassano R. N. Bradfield J. P. Sleiman P. M. Imielinski M. Guthery S. L. (2008). Loci on 20q13 and 21q22 are associated with pediatric-onset inflammatory bowel disease. *Nat. Genet.* 40 1211–1215. 10.1038/ng.203 18758464 PMC2770437

[B23] Laurent F. Mancassola R. Lacroix S. Menezes R. Naciri M. (2001). Analysis of chicken mucosal immune response to *Eimeria tenella* and *Eimeria* maxima infection by quantitative reverse transcription-PCR. *Infect. Immun.* 69 2527–2534. 10.1128/IAI.69.4.2527-2534.2001 11254616 PMC98188

[B24] Lee C. S. Hu C. Y. Tsai H. F. Wu C. S. Hsieh S. L. Liu L. C. (2008). Elevated serum decoy receptor 3 with enhanced T cell activation in systemic lupus erythematosus. *Clin. Exp. Immunol* 151 383–390. 10.1111/j.1365-2249.2007.03579.x 18190609 PMC2276966

[B25] Li W. Zhang C. Chen C. Zhuang G. (2007). Correlation between expression of DcR3 on tumor cells and sensitivity to FasL. *Cell Mol. Immunol.* 4 455–460. 18163957

[B26] Lillehoj H. S. Ding X. Quiroz M. A. Bevensee E. Lillehoj E. P. (2005). Resistance to intestinal coccidiosis following DNA immunization with the cloned 3-1E *Eimeria* gene plus IL-2, IL-15, and IFN-gamma. *Avian Dis.* 49 112–117. 10.1637/7249-073004R 15839423

[B27] Lima T. S. Gov L. Lodoen M. B. (2018). Evasion of human neutrophil-mediated host defense during *Toxoplasma gondii* infection. *mBio* 9:e02027-17. 10.1128/mBio.02027-17 29440572 PMC5821086

[B28] Lin W. W. Hsieh S. L. (2011). Decoy receptor 3: a pleiotropic immunomodulator and biomarker for inflammatory diseases, autoimmune diseases and cancer. *Biochem. Pharmacol.* 81 838–847. 10.1016/j.bcp.2011.01.011 21295012

[B29] Liu Y. L. Chen W. T. Lin Y. Y. Lu P. H. Hsieh S. L. Cheng I. H. (2017). Amelioration of amyloid-beta-induced deficits by DcR3 in an Alzheimer’s disease model. *Mol. Neurodegener.* 12:30 10.1186/s13024-017-0173-170 PMC5402663 28438208

[B30] Mabrouk I. Buart S. Hasmim M. Michiels C. Connault E. Opolon P. (2008). Prevention of autoimmunity and control of recall response to exogenous antigen by Fas death receptor ligand expression on T cells. *Immunity* 29 922–933. 10.1016/j.immuni.2008.10.007 19013083

[B31] Min W. Kim W. H. Lillehoj E. P. Lillehoj H. S. (2013). Recent progress in host immunity to avian coccidiosis: IL-17 family cytokines as sentinels of the intestinal mucosa. *Dev. Comp. Immunol.* 41 418–428. 10.1016/j.dci.2013.04.003 23583525

[B32] Min W. Lillehoj H. S. Burnside J. Weining K. C. Staeheli P. Zhu J. J. (2001). Adjuvant effects of IL-1beta, IL-2, IL-8, IL-15, IFN-alpha, IFN-gamma TGF-beta4 and lymphotactin on DNA vaccination against *Eimeria acervulina*. *Vaccine* 20 267–274. 10.1016/s0264-410x(01)00270-5 11567773

[B33] Morris G. M. Gasser R. B. (2006). Biotechnological advances in the diagnosis of avian coccidiosis and the analysis of genetic variation in *Eimeria*. *Biotechnol. Adv.* 24 590–603. 10.1016/j.biotechadv.2006.06.001 16901674

[B34] Pandori W. J. Lima T. S. Mallya S. Kao T. H. Gov L. Lodoen M. B. (2019). *Toxoplasma gondii* activates a Syk-CARD9-NF-kappaB signaling axis and gasdermin D-independent release of IL-1beta during infection of primary human monocytes. *PLoS Pathog.* 15:e1007923. 10.1371/journal.ppat.1007923 31449558 PMC6730955

[B35] Philip M. Rowley D. A. Schreiber H. (2004). Inflammation as a tumor promoter in cancer induction. *Semin. Cancer Biol.* 14 433–439. 10.1016/j.semcancer.2004.06.006 15489136

[B36] Pinard-Van D. L. M. Monvoisin J. L. Pery P. Hamet N. Thomas M. (1998). Comparison of outbred lines of chickens for resistance to experimental infection with coccidiosis (*Eimeria tenella*). *Poult. Sci.* 77 185–191. 10.1093/ps/77.2.185 9495476

[B37] Pitti R. M. Marsters S. A. Lawrence D. A. Roy M. Kischkel F. C. Dowd P. (1998). Genomic amplification of a decoy receptor for Fas ligand in lung and colon cancer. *Nature* 396 699–703. 10.1038/25387 9872321

[B38] Quiroz-Castaneda R. E. Dantan-Gonzalez E. (2015). Control of avian coccidiosis: future and present natural alternatives. *Biomed. Res. Int.* 2015:430610. 10.1155/2015/430610 25785269 PMC4346696

[B39] Rose M. E. Wakelin D. Hesketh P. (1991). Interferon-gamma-mediated effects upon immunity to coccidial infections in the mouse. *Parasite Immunol.* 13 63–74. 10.1111/j.1365-3024.1991.tb00263.x 1901641

[B40] Rothwell L. Young J. R. Zoorob R. Whittaker C. A. Hesketh P. Archer A. (2004). Cloning and characterization of chicken IL-10 and its role in the immune response to *Eimeria* maxima. *J. Immunol.* 173 2675–2682. 10.4049/jimmunol.173.4.2675 15294985

[B41] Sand J. M. Arendt M. K. Repasy A. Deniz G. Cook M. E. (2016). Oral antibody to interleukin-10 reduces growth rate depression due to *Eimeria* spp. infection in broiler chickens. *Poult. Sci.* 95 439–446. 10.3382/ps/pev352 26772659 PMC6592416

[B42] Sheng Z. Pettersson M. E. Hu X. Luo C. Qu H. Shu D. (2013). Genetic dissection of growth traits in a Chinese indigenous x commercial broiler chicken cross. *BMC Genomics* 14:151. 10.1186/1471-2164-14-151 23497136 PMC3679733

[B43] Shirley M. W. Smith A. L. Tomley F. M. (2005). The biology of avian *Eimeria* with an emphasis on their control by vaccination. *Adv. Parasitol.* 60 285–330. 10.1016/S0065-308X(05)60005-X 16230106

[B44] Song X. Huang X. Yan R. Xu L. Li X. (2015). Efficacy of chimeric DNA vaccines encoding *Eimeria tenella* 5401 and chicken IFN-gamma or IL-2 against coccidiosis in chickens. *Exp. Parasitol.* 156 19–25. 10.1016/j.exppara.2015.05.003 26008611

[B45] Tsai H. W. Huang M. T. Wang P. H. Huang B. S. Chen Y. J. Hsieh S. L. (2018). Decoy receptor 3 promotes cell adhesion and enhances endometriosis development. *J. Pathol.* 244 189–202. 10.1002/path.5000 29057478

[B46] Wakelin D. Rose M. E. Hesketh P. Else K. J. Grencis R. K. (1993). Immunity to coccidiosis: genetic influences on lymphocyte and cytokine responses to infection with *Eimeria* vermiformis in inbred mice. *Parasite Immunol.* 15 11–19. 10.1111/j.1365-3024.1993.tb00567.x 8094547

[B47] Yadav M. Clark L. Schorey J. S. (2006). Macrophage’s proinflammatory response to a mycobacterial infection is dependent on sphingosine kinase-mediated activation of phosphatidylinositol phospholipase C, protein kinase C, ERK1/2, and phosphatidylinositol 3-kinase. *J. Immunol.* 176 5494–5503. 10.4049/jimmunol.176.9.5494 16622018

[B48] Yang C. R. Hsieh S. L. Ho F. M. Lin W. W. (2005). Decoy receptor 3 increases monocyte adhesion to endothelial cells via NF-kappa B-dependent up-regulation of intercellular adhesion molecule-1, VCAM-1, and IL-8 expression. *J. Immunol.* 174 1647–1656. 10.4049/jimmunol.174.3.1647 15661928

[B49] Yun C. H. Lillehoj H. S. Lillehoj E. P. (2000). Intestinal immune responses to coccidiosis. *Dev. Comp. Immunol.* 24 303–324. 10.1016/s0145-305x(99)00080-4 10717295

[B50] Zhang X. Zhang Y. Xu J. Wang H. Zheng X. Lou Y. (2018). Antigen presentation of the Oct4 and Sox2 peptides by CD154-activated B lymphocytes enhances the killing effect of cytotoxic T lymphocytes on tumor stem-like cells derived from cisplatin-resistant lung cancer cells. *J Cancer* 9 367–374. 10.7150/jca.20821 29344283 PMC5771344

[B51] Zhang Y. Li D. Zhao X. Song S. Zhang L. Zhu D. (2015). Decoy receptor 3 suppresses FasL-induced apoptosis via ERK1/2 activation in pancreatic cancer cells. *Biochem. Biophys. Res. Commun.* 463 1144–1151. 10.1016/j.bbrc.2015.06.074 26102031

[B52] Zhang Y. Zhou Y. Sun G. Li K. Li Z. Su A. (2018). Transcriptome profile in bursa of Fabricius reveals potential mode for stress-influenced immune function in chicken stress model. *BMC Genomics* 19:918. 10.1186/s12864-018-5333-2 30545299 PMC6293626

[B53] Zheng X. Zhang B. Zhang Y. Zhong H. Nie R. Li J. (2020). Transcriptome analysis of feather follicles reveals candidate genes and pathways associated with pheomelanin pigmentation in chickens. *Sci. Rep.* 10:12088. 10.1038/s41598-020-68931-1 32694523 PMC7374586

[B54] Zhou J. Song S. He S. Wang Z. Zhang B. Li D. (2013). Silencing of decoy receptor 3 (DcR3) expression by siRNA in pancreatic carcinoma cells induces Fas ligand-mediated apoptosis in vitro and in vivo. *Int. J. Mol. Med.* 32 653–660. 10.3892/ijmm.2013.1437 23846297

