# Supplementary material for: Whole Transcriptome Analysis of Chicken Bursa Reveals Candidate Gene That Enhances the Host’s Immune Response to Coccidiosis
Source: Front Physiol. 2020 Oct 30;11:573676. doi: 10.3389/fphys.2020.573676 (PMC7662072; doi:10.3389/fphys.2020.573676)
Supplement: Supplementary Figure 1 — The lesion phenotypic and lesion score standard. [file Data_Sheet_1.PDF]

Supplementary Material

Supplementary Data

Supplementary Data 1. *TNFRSF6B* inhibits Fas signal pathway

The apoptosis of immune cells is a kind of process which affects the immune response. Therefore, we also explored the effect of *TNFRSF6B* on macrophages apoptosis. Flow cytometry was performed to measure the macrophages apoptosis. The result showed a negative role of *TNFRSF6B* on HD11 cells apoptosis (Supplementary Figure 2a, b). In this regard, we proposed a hypothesis that *TNFRSF6B* would induce anti-apoptosis in macrophages via regulating Fas signal and the immunity activity would therefore be enhanced. To investigate this hypothesis, we conducted a series of experiments. Fas signaling pathway tends to result in the losing integral of nuclear membrane and low synthesis of poly. We detected the mRNA expression of related genes, including *PARP1*, *PARP3* and *Lamin B2* (Supplementary Figure 2c, d). The protein levels of PARP1 and Lamin B were also measured by western blot (Supplementary Figure 2g-j). The results fully show a positive effect of *TNFRSF6B* on antagonizing macrophage apoptosis.

In addition, we also detected the mRNA expression of core genes in Fas signaling pathway, including *FADD*, *CASP3*, *CASP6*, *CASP7*, *CASP8*, *CASP9*, *CASP10*, *Cyt c* and *Fas* (Supplementary Figure 2e, f). It is showed that *TNFRSF6B* overexpression significantly downregulates their mRNA expression, while knockdown of *TNFRSF6B* upregulates them. Moreover, the protein levels of Cleaved-Caspase 8 and Cleaved-Caspase 9 were decreased dramatically (Supplementary Figure 2g-j). Totally, these results imply that *TNFRSF6B* can downregulate Fas signal pathway, thus inhibiting the apoptosis of macrophages.

Supplementary Tables

Supplementary Table 1. Primers used for qPCR

Primer name	Sequences (5'-3')	Product size (bp)	Annealing temperature (◦C)
q-TNFRSF6B-F	GGCTCCGGCGTCGTGAAGTT	177	59
q-TNFRSF6B-R	GCACAGGGTGTCGTGGTATTG		
q-IL-1β-F	AAGAGACCTTCTACGGCCCC	233	60
q-IL-1β-R	GACGGGCTCAAAAACCTCCT		
q-IL-2-F	TCTGGGACCACTGTATGCTCT	63	60
q-IL-2-R	ACACCAGTGGGAAACAGTATCA		
q-IL-6-F	CAAGGTGACGGAGGAGGAC	254	60
q-IL-6-R	TGGCGAGGAGGGATTTCT		
q-IL-8-F	GGCTTGCTAGGGGAAATGA	200	60
q-IL-8-R	AGCTGACTCTGACTAGGAAACTGT		

q-IL-10-F	CGGGAGCTGAGGGTGAA	272	60
q-IL-10-R	GTGAAGAAGCGGTGACAGC		
q-IL-12-F	AGACTCCAATGGGCAAATGA	264	60
q-IL-12-R	CTCTTCGGCAAATGGACAGT		
q-IFN-γ-F	GACATCCTTCAGCATCTCTTCA	238	60
q-IFN-γ-R	AGGCGCTGTAATCGTTGTCT		
q-TNF-α-F	AGCAGGGCTGACACGGAT	338	60
q-TNF-α-R	TGTTGGCATAGGCTGTCCTG		
q-CSF-1-F	GGCACAGTGTCCTTCAGATTC	245	58
q-CSF-1-R	AACTCCTCAAAGCACATTTCG		
q-CSF-2-F	GTCTGCGTAACAACCTGA	62	52
q-CSF-2-R	TTCTTCCTCTGTCCCATT		
q-TLR2A-F	GACCTTCTGCACTCTGCCAT	229	60
q-TLR2A-R	ATGCTCCTTTCCCCTCAAGC		
q-TLR2B-F	TAGTGGCCATGTCGATCAGC	273	60
q-TLR2B-R	AGCAGATGTCTTTCGTGGGG		
q-TLR4-F	TTCGGTTGGTGGACCTGAAT	258	59
q-TLR4-R	TAGGGGAACAGCAGGTAAGGA		
q-CASP3-F	TGGCCCTCTTGAACTGAAAG	139	61
q-CASP3-R	TCCACTGTCTGCTTCAATACC		
q-CASP6-F	TAGACGTGGGACTTTGGCAG	309	61
q-CASP6-R	TCACCTCGACATGCCTGAAT		
q-CASP7-F	GGGTACACGCAATGGAACTG	312	60
q-CASP7-R	CCTCGGCATGCCTGAATGAA		
q-CASP8-F	CCCTGAAGACAGTGCCATTT	106	61
q-CASP8-R	GGGTCGGCTGGTCATTTTAT		
q-CASP9-F	TCCCGGGCTGTTTCAACTT	61	61
q-CASP9-R	CCTCATCTTGCAGCTTGTGC		
q-CASP10-F	GCCTAGGTGACATCAAGCCC	270	60
q-CASP10-R	CCATGGAGCCGTAAGTCCAA		
q-Fas-F	TCCACCTGCTCCTCGTCATT	78	61
q-Fas-R	GTGCAGTGTGTGTGGGAACT		
q-Cyt C-F	TGTCCAGAAATGTTCCCAGTGC	138	61
q-Cyt C-R	CCTTTGTTCTTATTGGCATCTGTG		
q-FADD-F	CTGCGAGCTGAAGTTTCTGTG	105	60
q-FADD-R	ATCAGTTGCTGCTCCATGAGG		
q-PRAP1-F	AGGCTATTGAAACCGGAGGC	231	58
q-PRAP1-R	AGTCCGGGTGGTACCAGTTA		

q-PRAP3-F	CTTGGACCTCTCAAACCGCT	392	60
q-PRAP3-R	GCAGCTGGCGATTATGAAGC		
q-Lamin B2-F	CCGTCTATATCGACCGCGTG	274	60
q-Lamin B2-R	ATGCGACCTTGAGCAACAGA		
q-GAPDH-F	TCCTCCACCTTTGATGCG	225	50-65
q-GAPDH-R	GTGCCTGGCTCACTCCTT		

Supplementary Table 2. Data statistics.

Sample name	Pair-end reads	Clean bases	GC Content	%≥Q30
BAE1	31,281,495	9,344,553,636	52.33%	94.51%
BAE2	36,491,314	10,895,831,630	52.65%	94.66%
BAE3	34,278,860	10,241,490,772	52.18%	94.56%
BAN1	33,371,431	9,971,924,464	52.46%	94.75%
BAN2	42,143,806	12,603,480,938	52.18%	94.66%
BAN3	37,408,273	11,179,970,460	52.17%	94.59%

Supplementary Table 3. Clean reads mapped efficiency.

Sample name	Total clean reads	Mapped reads	Uniq mapped reads	Multiple map reads	Reads map to '+'	Reads map to '-'
BAE1	62,562,990	56,335,799 (90.05%)	53,897,183 (86.15%)	2,438,616 (3.90%)	27,832,715 (44.49%)	27,910,674 (44.61%)
BAE 2	72,982,628	64,839,190 (88.86%)	61,904,315 (84.82%)	2,934,839 (4.02%)	31,984,438 (43.82%)	32,104,354 (43.99%)
BAE 3	68,557,720	61,653,469 (89.93%)	58,974,215 (86.02%)	2,679,254 (3.91%)	30,467,398 (44.44%)	30,560,711 (44.58%)
BAN1	66,742,862	59,144,563 (88.62%)	56,384,926 (84.48%)	2,759,637 (4.13%)	29,762,845 (43.69%)	29,271,507 (43.86%)
BAN2	84,287,612	76,770,262 (89.89%)	72,474,871 (85.99%)	3,295,391 (3.91%)	37,435,690 (44.41%)	37,547,416 (44.55%)
BAN3	74,816,546	67,335,604 (90.00%)	64,390,520 (86.06%)	2,945,084 (3.94%)	33,259,571 (44.45%)	33,361,799 (44.59%)

Supplementary Table 4. Gene count statistics in RNA sequencing.

Sample name	Gene number	Known gene number	Novel gene number

BAE1	18,462	15,882	2,580
BAE 2	18,553	15,844	2,609
BAE 3	18,501	15,872	2,629
BAN1	18,485	15,872	2,613
BAN2	18,675	15,990	2,685
BAN3	18,475	15,882	2,593
Total	19,866	16,231	3,635

Supplementary Table 5. DEGs between BAE and BAN

Gene_name	BAE_FPKM	BAN_FPKM	P-value	log2FC	Regulated
CATHB1	21055.78	11860.87	0.000027	0.821464	up
RARRES1	1910.72	1055.34	0.000072	0.847931	up
TF	438.25	209.09	0.000004	1.062597	up
LCN8	328.32	120.76	0.001096	1.424024	up
KRT19	129.41	84.88	0.004551	0.598259	up
LDHA	117.84	71.60	0.000038	0.704872	up
AVD	115.05	43.37	0.000106	1.403992	up
SCCPDH	77.24	46.77	0.000215	0.711753	up
LOC107053341	26.50	96.59	0.000090	-1.89748	down
LOC107048987	67.63	36.31	0.005604	0.886927	up
DPYSL3	32.18	62.42	0.000220	-0.98482	down
RP11-295K3.1	57.19	36.46	0.017865	0.63303	up
VPS29	54.06	33.96	0.001172	0.726289	up
ASS1	53.46	34.01	0.017509	0.636107	up
MRPS10	27.43	53.81	0.021743	-0.94719	down
AGR2	59.27	21.13	0.000000	1.4751	up
PELI1	27.46	44.33	0.001300	-0.64404	down
TRPV2	48.21	19.87	0.000023	1.086721	up
LOC769384	15.03	46.85	0.046737	-1.51015	down
ATP1B1	36.06	20.97	0.004076	0.767551	up
TLR1A	32.75	23.46	0.000312	0.959877	up
LOC427029	34.27	20.93	0.012347	0.591976	up
MTHFD2	20.08	33.82	0.010891	-0.76253	down
PLVAP	31.85	20.95	0.018888	0.593018	up
LOC107054036	36.96	15.75	0.049778	0.81583	up
ACOT9	20.05	30.52	0.005593	-0.61058	down
NOXO1	29.56	17.07	0.016887	0.795457	up
APOC3	34.00	11.73	0.007138	1.530247	up
SULT1B1	28.10	16.61	0.009748	0.786807	up
LOC107051274	25.92	16.88	0.014697	0.961958	up
LOC429682	16.85	24.99	0.038597	-0.62172	down
TUBB2A	24.40	17.18	0.015063	0.627392	up
ATP11C	24.25	16.73	0.009971	0.614172	up
LOC107052256	14.42	25.34	0.029179	-0.82489	down

LOC417253	20.90	12.95	0.014825	0.664413	up
SMEK2	11.72	21.95	0.000001	-0.87349	down
RNF223	14.08	19.48	0.000066	-0.68846	down
LOC107056758	12.75	20.01	0.038389	-0.66416	down
ECM1	22.38	9.81	0.005787	1.169704	up
FAM193B	13.34	18.33	0.013419	-0.59851	down
PMS1	16.52	14.29	0.000068	0.784447	up
PEBP4	19.93	10.42	0.015506	0.942362	up
ST3GAL5	10.24	19.82	0.009023	-0.93541	down
ACTR10L	11.48	18.45	0.011216	-0.71339	down
HDGFRP3	17.89	12.00	0.018227	0.599343	up
LOC107057400	14.19	14.62	0.024556	-0.69069	down
C10H15ORF26	9.67	17.81	0.002528	-0.68019	down
MLEC	10.13	17.31	0.004103	-0.82235	down
GPX2	18.85	8.34	0.005993	1.161093	up
COMTD1	16.71	10.23	0.048623	0.756359	up
LOC107049471	24.11	2.36	0.001276	2.602104	up
ASL2	17.70	7.50	0.007458	1.369382	up
ORC1	9.87	15.23	0.001553	-2.53771	down
LOC107055521	23.29	1.64	0.000857	3.977346	up
TAPT1	15.56	9.26	0.020876	0.798956	up
LOC425783	18.08	6.59	0.046112	0.708209	up
LOC107054816	8.69	13.97	0.043084	-0.74742	down
RP5-877J2.1	14.10	8.37	0.039107	0.712086	up
TMED3	14.04	8.11	0.040177	0.77734	up
IGFBP4	13.26	8.75	0.049127	0.592045	up
RAB27A	13.45	7.99	0.023309	0.801761	up
ATP12A	12.74	7.78	0.037184	0.701164	up
SLC39A8	12.97	7.41	0.022926	0.664579	up
LOC100859655	11.89	8.46	0.000032	1.84019	up
CD200	11.99	7.51	0.011725	0.758052	up
TNFRSF6B	14.22	5.13	0.000134	1.408344	up
LOC107049382	18.83	0.11	0.002294	6.618017	up
FGFBP1	13.05	5.69	0.014902	1.212838	up
NANP	10.67	7.97	0.015394	0.66635	up
CHAC1	4.21	14.39	0.000576	-1.79618	down
LOC100858664	11.48	6.98	0.027895	0.665949	up
CHIA	12.53	5.84	0.048929	1.051355	up
RRBP1	10.87	7.19	0.017317	0.613554	up
UGT2A1	10.91	6.47	0.034043	0.732307	up
CGGBP1	13.10	4.10	0.000000	1.839408	up
ARL8BL	4.89	11.59	0.020320	-1.24816	down
GAL3ST2	11.14	4.98	0.012299	1.161813	up
NADK2	6.23	9.77	0.036938	-0.74447	down
LOC769755	5.67	10.31	0.049773	-0.68459	down
SLC34A2	10.50	5.41	0.005261	0.900787	up
RCHY1	13.15	2.11	0.001273	2.63675	up
SLC7A5	5.82	9.09	0.040139	-0.65203	down

SLC26A5	9.75	5.05	0.000230	0.931904	up
SLC6A14	9.20	5.48	0.030116	0.740838	up
ALG13	9.05	5.53	0.042127	0.642147	up
LOC101747455	9.07	5.19	0.039017	0.807805	up
LOC107054223	0.06	14.13	0.000000	-7.45697	down
CELSR1	8.76	5.35	0.014199	0.756611	up
LOC107050209	9.10	4.61	0.004011	1.097125	up
GTF2E2	10.57	3.08	0.000697	1.795603	up
MAP6D1	9.11	4.51	0.008305	0.910279	up
LOC107056127	0.90	12.47	0.001050	-3.78047	down
LOC107050060	10.67	2.70	0.006413	2.372956	up
PLBD1	8.98	3.93	0.001758	1.099523	up
POLG2	4.22	8.67	0.009566	-0.97039	down
MTPAP	4.65	8.14	0.022115	-0.58776	down
LOC101748375	3.78	8.65	0.011584	-1.2897	down
THAP9	8.83	3.49	0.006675	1.248935	up
KCNJ16	7.13	4.98	0.045861	0.607162	up
CTDSPL	5.08	6.94	0.003896	-1.01939	down
HBG2	1.53	10.30	0.000266	-2.69509	down
STK3	6.78	4.67	0.039862	0.5943	up
MAX	3.22	8.04	0.026951	-1.31687	down
PIKFYVE	6.47	4.72	0.003731	0.730262	up
LOC100858620	2.55	8.63	0.009397	-1.58553	down
CPM	6.99	4.08	0.023592	0.76083	up
LOC107051015	7.64	3.29	0.006378	1.254103	up
CYLD	7.41	3.46	0.000135	1.095461	up
LOC100857553	2.72	8.11	0.000170	-1.5434	down
LOC107056855	10.38	0.36	0.000000	4.781713	up
NOV	6.89	3.76	0.045205	0.842655	up
CWH43	7.09	3.52	0.008288	1.077033	up
LOC423849	6.71	3.88	0.030616	0.731243	up
FZD5	6.65	3.83	0.044140	0.778713	up
PLEKHG5	6.47	3.91	0.021567	0.712287	up
TAGLN	1.27	9.04	0.000027	-2.8129	down
ZNF568	3.39	6.75	0.012855	-0.79073	down
HAUS3	6.13	3.97	0.019798	0.609084	up
PAM	6.23	3.76	0.037124	0.709935	up
LOC425534	7.76	2.22	0.011806	1.916944	up
NEU3	5.83	4.09	0.006331	1.222394	up
RALGPS2	5.86	4.04	0.000064	0.998167	up
LOC419276	6.65	3.06	0.006566	1.105564	up
LOC107056755	0.96	8.72	0.000695	-3.15427	down
MGA	3.69	5.97	0.011014	-0.80705	down
KIAA0513	6.20	3.41	0.028952	0.817631	up
EPS15L1	5.58	3.56	0.001242	0.903063	up
LOC101750739	6.70	2.22	0.011659	1.546419	up
KLHL17	2.95	5.91	0.009072	-0.75212	down

TLR1B	6.38	2.28	0.000464	1.721404	up
LOC107056145	5.88	2.71	0.040331	0.91852	up
BHLHE23	3.02	5.39	0.017319	-0.82477	down
SCNN1B	2.72	5.68	0.046719	-1.05372	down
PANX3	2.75	5.62	0.049995	-1.01248	down
LOC107057037	1.20	7.06	0.000564	-2.07057	down
SLC2A13	3.12	5.12	0.039287	-0.77809	down
SMUG1	5.46	2.74	0.038021	0.974044	up
C1H11ORF82	2.83	5.28	0.031068	-0.85656	down
CD24	5.25	2.79	0.033443	0.90087	up
WWC1	2.88	5.15	0.019023	-0.97497	down
RBM41	0.64	7.36	0.000087	-3.49909	down
SEC24D	4.75	3.20	0.045049	0.656375	up
LOC107057197	0.56	7.31	0.000000	-3.772	down
DCP2	5.12	2.70	0.006089	0.883278	up
LOC100859694	6.51	1.26	0.025533	2.314863	up
POLN	2.65	4.99	0.001716	-0.98385	down
AMHR2	5.52	1.90	0.018432	1.288115	up
LOC107054766	4.49	2.91	0.046843	0.728188	up
EPS8	4.53	2.83	0.045360	0.704392	up
FCHO2	4.92	2.26	0.002563	1.096571	up
LOC107052719	5.22	1.90	0.003234	1.430418	up
TTC25	2.05	5.02	0.005068	-1.62617	down
LOC101747844	4.35	2.58	0.018102	0.790352	up
LOC430731	4.81	2.07	0.035914	1.078361	up
LAMP3	4.51	2.35	0.004766	2.07554	up
LOC107053337	4.42	2.34	0.039226	0.989963	up
LOC770556	4.63	2.09	0.005020	1.090164	up
CTBP2	4.29	2.34	0.025763	0.837814	up
P2RY11	4.87	1.72	0.023946	1.453346	up
ELF5	4.33	2.23	0.023081	0.906384	up
KDELR3	4.46	2.08	0.011899	1.087442	up
ACTG2	0.38	6.03	0.000001	-3.95543	down
VWA2	4.71	1.67	0.000341	1.488608	up
LOC107055757	5.31	0.95	0.002027	2.448618	up
LOC107057415	3.98	2.11	0.000133	1.244354	up
ENPP2	3.98	2.07	0.031137	0.996561	up
C2ORF88	3.84	2.15	0.015357	0.94046	up
GLIS2	4.00	1.96	0.008872	1.082109	up
VDAC3	4.10	1.85	0.033914	1.085351	up
LOC100859202	1.18	4.61	0.001187	-1.75791	down
DACT2	1.83	3.89	0.030916	-1.23889	down
GRHL1	3.47	2.17	0.035829	0.984278	up
CLDN4	3.76	1.85	0.046685	1.005683	up
LOC107049124	1.48	4.12	0.009058	-1.50266	down
LOC107057031	3.63	1.93	0.038735	0.971917	up
CNN1	1.06	4.43	0.003246	-2.12133	down
VWA5B2	1.49	3.96	0.010868	-1.47666	down

LOC107057220	3.88	1.55	0.044452	1.195748	up
LOC107051647	3.47	1.79	0.036435	1.095147	up
TTC33	4.16	1.08	0.000124	2.060616	up
LOC422150	1.66	3.55	0.029440	-0.95547	down
IL10	4.87	0.29	0.000000	3.764468	up
GPR37	1.20	3.90	0.009584	-1.65976	down
HNF4beta	1.58	3.42	0.013641	-1.2295	down
SLC7A10	1.96	3.04	0.049240	-0.63791	down
BPI	3.30	1.69	0.033704	0.976979	up
TNRC6B	3.24	1.69	0.000191	1.042071	up
LIMK2	3.06	1.86	0.046198	0.748162	up
STX5	4.03	0.86	0.009780	1.935809	up
TNFRSF25	3.20	1.66	0.028197	0.978201	up
METTL7A	3.30	1.55	0.048781	1.061532	up
TIE1	2.86	1.90	0.041421	0.631779	up
LOC107051466	4.03	0.70	0.031202	2.578436	up
SLC2A9	2.98	1.70	0.024511	0.811708	up
SELPLG	3.66	0.95	0.003444	1.91456	up
LOXL2	3.93	0.59	0.000000	2.729772	up
PFKFB4	1.18	3.26	0.003878	-1.53144	down
LOC107052134	1.33	3.05	0.026804	-1.19301	down
FAM217B	2.63	1.75	0.014796	0.955143	up
HTRA3	2.77	1.48	0.038862	0.891252	up
FSBP	2.44	1.76	0.021100	0.911433	up
SLC16A3	1.07	3.13	0.008080	-1.54895	down
KRT20	2.80	1.34	0.046956	1.077433	up
TREM-B1	2.82	1.31	0.037779	1.173711	up
KCNJ15	2.47	1.50	0.045710	0.965488	up
APOOL	2.56	1.40	0.019682	3.182518	up
CDKN2B	2.78	1.14	0.044936	1.154623	up
PPM1F	2.19	1.71	0.002467	1.207427	up
LOC107054764	2.80	1.07	0.010365	1.597682	up
FAM89A	1.22	2.55	0.049655	-0.942	down
TNC	1.01	2.71	0.004664	-1.17411	down
LOC107051636	2.62	1.07	0.009046	1.4722	up
PPAPDC1B	2.93	0.75	0.025278	1.613221	up
LOC429249	2.44	1.23	0.028197	0.986101	up
LOC107056495	2.79	0.82	0.016042	1.748984	up
LOC107049971	0.64	2.95	0.015040	-2.15698	down
ZNF395	1.28	2.21	0.040870	-0.81773	down
MYO5C	2.35	1.14	0.032866	0.933421	up
GGT1	2.30	1.08	0.027283	1.05879	up
ZNF148	2.14	0.96	0.002527	1.611967	up
LOC107049272	0.62	2.39	0.011538	-1.96217	down
LOC107049190	0.84	2.12	0.028935	-1.16083	down
LOC107054696	0.80	2.14	0.030511	-1.41909	down
TAAR1	0.00	2.90	0.000000	-8.25323	down

TMEM2	2.40	0.50	0.000124	2.300358	up
SEMA3B	1.95	0.92	0.025297	1.197092	up
LOC107057417	1.87	0.95	0.017583	1.88738	up
CD101	2.08	0.74	0.006110	1.538384	up
GRHL2	1.77	0.99	0.046568	0.890634	up
LOC107049100	1.67	1.03	0.013360	1.529217	up
LOC107051489	1.90	0.77	0.028688	1.294999	up
C9H2ORF72	0.87	1.80	0.046967	-1.0519	down
LOC107050575	1.02	1.62	0.007120	-1.74346	down
ESPN	1.72	0.88	0.039054	1.138535	up
NT5E	1.67	0.92	0.045080	0.952376	up
LOC101748058	0.44	2.14	0.000010	-2.63791	down
LOC107056699	1.80	0.67	0.013881	1.403323	up
LOC107056556	0.74	1.72	0.032030	-1.21825	down
MARCO	1.80	0.65	0.029785	1.449787	up
SLC13A2	1.63	0.79	0.036773	1.097536	up
RANBP3L	1.66	0.69	0.009922	1.277887	up
KCNS1	0.51	1.82	0.006854	-1.66692	down
CMBL	1.81	0.48	0.004952	1.857615	up
LOC107052718	2.23	0.05	0.000000	5.379923	up
MCCC2	2.18	0.08	0.000064	4.799819	up
LOC100858447	0.71	1.51	0.036110	-1.14956	down
ADAM8	1.50	0.72	0.036567	1.118353	up
CBX6	0.72	1.49	0.026426	-1.04812	down
KCNS2	0.35	1.84	0.028696	-1.87722	down
AVPR1B	1.67	0.51	0.028880	1.687287	up
SLCO4C1	1.59	0.56	0.034389	1.216815	up
NTRK1	0.50	1.65	0.004840	-1.6073	down
LOC101748144	1.40	0.75	0.031916	1.255809	up
TFPI	1.42	0.69	0.043682	1.09073	up
LOC101750175	1.56	0.53	0.014184	1.433921	up
NEURL1	0.40	1.68	0.000073	-2.05658	down
PPARG	1.28	0.66	0.022549	1.159837	up
GNAQ	1.17	0.69	0.046013	0.996212	up
STX3	1.29	0.52	0.009774	1.478554	up
FHL5	1.40	0.35	0.019667	1.399442	up
GALNT5	1.16	0.54	0.048774	1.033597	up
LOC107057314	1.37	0.33	0.002446	1.970254	up
LOC107057521	0.28	1.38	0.000232	-2.2766	down
VIT	1.31	0.29	0.026455	1.714497	up
LOC101750842	1.07	0.42	0.043670	1.172187	up
GJB2	0.36	1.09	0.044869	-1.53617	down
IL12RB2	0.89	0.56	0.003060	1.426233	up
TTLL4	0.20	1.25	0.020437	-2.52244	down
SYCP2	0.33	1.11	0.042359	-1.40158	down
LOC771494	1.05	0.39	0.005914	1.192149	up
FEV	1.20	0.22	0.000327	2.351393	up
SLC38A3	0.98	0.42	0.028084	1.317883	up

FUT5	0.96	0.42	0.027350	1.178016	up
LOC425792	0.50	0.84	0.043498	-1.1539	down
IL1R1	0.97	0.37	0.031428	1.367466	up
EDNRB2	0.79	0.54	0.045414	1.048995	up
ZSWIM3	0.95	0.32	0.008636	1.542197	up
LOC100857180	0.97	0.29	0.006208	1.639096	up
LOC418189	0.16	1.08	0.000585	-2.14606	down
SOUL	0.29	0.85	0.023120	-1.57052	down
RAPGEFL1	0.31	0.78	0.024623	-1.34861	down
ETV4	0.87	0.20	0.005726	1.941239	up
TTLL2	0.32	0.75	0.048871	-1.288	down
TBX1	0.70	0.33	0.010401	1.147037	up
LOC415478	0.26	0.74	0.030079	-1.25695	down
KCNK12	0.70	0.26	0.046172	1.231407	up
CEMIP	0.29	0.66	0.044969	-1.14797	down
LOC101751375	0.77	0.12	0.016906	2.11037	up
LRRC34	0.65	0.21	0.046228	1.560766	up
CHAT2	0.68	0.12	0.046416	1.875122	up
LOC107055070	0.62	0.18	0.019337	1.728962	up
LOC101751598	0.18	0.48	0.036690	-1.53231	down
LOC101750894	0.43	0.18	0.030082	1.213936	up
LOC101748210	0.11	0.50	0.000964	-2.16036	down
CFTR	0.36	0.14	0.024698	1.358559	up
ISM1	0.32	0.04	0.033687	1.290979	up
CREB5	0.19	0.11	0.048233	1.15376	up
ADRA2C	0.02	0.13	0.007534	-2.38273	down
Gallus_gallus_newGene_1	131.98	281.70	0.000074	-1.33248	down
Gallus_gallus_newGene_5066	49.62	135.05	0.000260	-1.89371	down
Gallus_gallus_newGene_14072	58.65	19.81	0.029042	2.14076	up
Gallus_gallus_newGene_7348	31.73	15.44	0.000002	1.025297	up
Gallus_gallus_newGene_8559	4.76	10.99	0.006884	-1.15439	down
Gallus_gallus_newGene_10303	9.31	3.29	0.024818	1.499718	up
Gallus_gallus_newGene_15851	2.49	7.22	0.019179	-1.51562	down
Gallus_gallus_newGene_16210	1.89	6.04	0.005830	-1.52846	down
Gallus_gallus_newGene_15516	6.33	0.12	0.000000	5.504896	up
Gallus_gallus_newGene_3604	5.92	0.49	0.000154	3.549519	up
Gallus_gallus_newGene_6760	4.27	1.80	0.007913	1.373617	up
Gallus_gallus_newGene_15312	1.26	4.59	0.004148	-1.63542	down
Gallus_gallus_newGene_18320	3.85	1.44	0.004350	1.403748	up
Gallus_gallus_newGene_14723	1.16	4.00	0.000278	-1.78986	down
Gallus_gallus_newGene_16504	1.19	3.62	0.001298	-1.89933	down
Gallus_gallus_newGene_10321	3.19	1.61	0.029613	0.820012	up
Gallus_gallus_newGene_17599	0.01	4.79	0.000000	-8.40213	down
Gallus_gallus_newGene_15405	1.36	3.33	0.031015	-1.28652	down
Gallus_gallus_newGene_14718	4.01	0.64	0.000034	3.618685	up
Gallus_gallus_newGene_17616	1.05	3.28	0.034441	-1.4417	down
Gallus_gallus_newGene_1369	3.03	0.82	0.000062	1.889295	up

Gallus_gallus_newGene_17213	1.34	2.42	0.011693	-1.06357	down
Gallus_gallus_newGene_15792	2.95	0.50	0.001123	2.765388	up
Gallus_gallus_newGene_13941	0.48	2.95	0.000010	-2.65119	down
Gallus_gallus_newGene_3327	0.28	3.13	0.008558	-3.62734	down
Gallus_gallus_newGene_17861	0.88	2.49	0.029969	-1.6002	down
Gallus_gallus_newGene_3304	0.82	2.37	0.025803	-1.47631	down
Gallus_gallus_newGene_9297	0.65	2.48	0.013598	-1.85953	down
Gallus_gallus_newGene_15794	0.66	2.25	0.017640	-1.75096	down
Gallus_gallus_newGene_13364	1.71	0.76	0.007637	1.525194	up
Gallus_gallus_newGene_12626	0.44	1.91	0.024862	-2.11028	down
Gallus_gallus_newGene_9674	1.58	0.66	0.025557	1.235936	up
Gallus_gallus_newGene_13424	0.04	2.12	0.000000	-5.67937	down
Gallus_gallus_newGene_7378	0.56	1.56	0.008413	-1.84717	down
Gallus_gallus_newGene_15594	1.80	0.30	0.000527	2.528407	up
Gallus_gallus_newGene_17373	0.51	1.48	0.014648	-1.52516	down
Gallus_gallus_newGene_20551	1.42	0.56	0.020085	1.172752	up
Gallus_gallus_newGene_12167	0.65	1.17	0.032699	-0.98903	down
Gallus_gallus_newGene_13376	1.34	0.40	0.002589	2.096693	up
Gallus_gallus_newGene_2630	0.39	1.30	0.018171	-1.3149	down
Gallus_gallus_newGene_1982	1.42	0.26	0.003943	2.358408	up
Gallus_gallus_newGene_15726	1.47	0.10	0.000000	3.751812	up
Gallus_gallus_newGene_16914	0.03	1.43	0.000003	-5.49627	down
Gallus_gallus_newGene_13326	1.13	0.25	0.009169	2.189289	up
Gallus_gallus_newGene_19324	0.62	0.25	0.034471	1.165486	up
Gallus_gallus_newGene_95	0.00	0.47	0.000000	-10.3174	down
Gallus_gallus_newGene_8805	0.35	0.10	0.013776	1.896423	up
Gallus_gallus_newGene_12295	0.33	0.10	0.013132	1.65221	up
Gallus_gallus_newGene_7931	0.29	0.10	0.046353	1.512362	up

Supplementary Table 6. GO enrichment for DEGs.

#Domain	GO_ID	Term	Annotated	Significant	Expected	KS
Biological Process	GO:0006278	RNA-dependent DNA biosynthetic process	78	1	1.22	1.00E-30
Biological Process	GO:0002474	antigen processing and presentation of peptide antigen via MHC class I	40	2	0.63	1.30E-11
Biological Process	GO:0001916	positive regulation of T cell mediated cytotoxicity	32	2	0.5	5.10E-10
Biological Process	GO:0007156	homophilic cell adhesion via plasma membrane adhesion molecules	107	1	1.67	1.6E-06
Biological Process	GO:0000245	spliceosomal complex assembly	22	1	0.34	5.8E-06
Biological Process	GO:2001243	negative regulation of intrinsic apoptotic signaling pathway	13	1	0.2	0.000022
Biological Process	GO:0031134	sister chromatid biorientation	6	1	0.09	0.000044
Biological Process	GO:0071922	regulation of cohesin loading	8	1	0.13	0.0001
Biological Process	GO:0071459	protein localization to chromosome, centromeric region	7	1	0.11	0.00024
Biological Process	GO:0097284	hepatocyte apoptotic process	7	1	0.11	0.00024
Biological Process	GO:0071930	negative regulation of transcription involved in G1/S transition of mitotic cell cycle	7	1	0.11	0.00024

Biological Process	GO:0034349	glial cell apoptotic process	8	1	0.13	0.00034
Biological Process	GO:0000819	sister chromatid segregation	55	1	0.86	0.00059
Biological Process	GO:0045842	positive regulation of mitotic metaphase/anaphase transition	8	1	0.13	0.00087
Biological Process	GO:0000082	G1/S transition of mitotic cell cycle	68	3	1.06	0.00095
Biological Process	GO:0010745	negative regulation of macrophage derived foam cell differentiation	8	1	0.13	0.00109
Biological Process	GO:0045651	positive regulation of macrophage differentiation	11	1	0.17	0.00152
Biological Process	GO:0016032	viral process	65	1	1.02	0.00166
Biological Process	GO:0050821	protein stabilization	34	1	0.53	0.00176
Biological Process	GO:1902115	regulation of organelle assembly	23	1	0.36	0.00213
Biological Process	GO:0034767	positive regulation of ion transmembrane transport	8	1	0.13	0.00329
Biological Process	GO:2000134	negative regulation of G1/S transition of mitotic cell cycle	21	3	0.33	0.00343
Biological Process	GO:0097191	extrinsic apoptotic signaling pathway	29	1	0.45	0.00365
Biological Process	GO:0021591	ventricular system development	8	1	0.13	0.00373
Biological Process	GO:0045879	negative regulation of smoothened signaling pathway	20	1	0.31	0.00386
Biological Process	GO:0034088	maintenance of mitotic sister chromatid cohesion	10	1	0.16	0.00509
Biological Process	GO:0021884	forebrain neuron development	16	1	0.25	0.00612
Biological Process	GO:0007067	mitotic cell cycle	185	3	2.89	0.00642
Biological Process	GO:0022604	regulation of cell morphogenesis	157	2	2.46	0.00656
Biological Process	GO:0033363	secretory granule organization	7	1	0.11	0.00777
Biological Process	GO:0018200	peptidyl-glutamic acid modification	8	1	0.13	0.00808
Biological Process	GO:0019882	antigen processing and presentation	66	2	1.03	0.00832
Biological Process	GO:0042326	negative regulation of phosphorylation	134	2	2.1	0.00937
Biological Process	GO:0046415	urate metabolic process	8	1	0.13	0.01026
Biological Process	GO:0002228	natural killer cell mediated immunity	12	1	0.19	0.01171
Biological Process	GO:0042267	natural killer cell mediated cytotoxicity	12	1	0.19	0.01171
Biological Process	GO:0007266	Rho protein signal transduction	96	1	1.5	0.01295
Biological Process	GO:0050709	negative regulation of protein secretion	27	1	0.42	0.01484
Biological Process	GO:0043353	enucleate erythrocyte differentiation	12	1	0.19	0.01564
Biological Process	GO:0051648	vesicle localization	62	1	0.97	0.01784
Biological Process	GO:0009084	glutamine family amino acid biosynthetic process	14	3	0.22	0.01912
Biological Process	GO:2000045	regulation of G1/S transition of mitotic cell cycle	34	3	0.53	0.0192
Biological Process	GO:1902806	regulation of cell cycle G1/S phase transition	34	3	0.53	0.0192
Biological Process	GO:0070647	protein modification by small protein conjugation or removal	310	3	4.85	0.02012
Biological Process	GO:0042787	protein ubiquitination involved in ubiquitin-dependent protein catabolic process	32	1	0.5	0.02018
Biological Process	GO:0007350	blastoderm segmentation	8	1	0.13	0.02072
Biological Process	GO:2001236	regulation of extrinsic apoptotic signaling pathway	20	1	0.31	0.02206
Biological Process	GO:0046467	membrane lipid biosynthetic process	43	1	0.67	0.02231
Biological Process	GO:1990138	neuron projection extension	30	1	0.47	0.02289
Biological Process	GO:0048675	axon extension	30	1	0.47	0.02289
Biological Process	GO:0045649	regulation of macrophage differentiation	18	2	0.28	0.02434
Biological Process	GO:0045650	negative regulation of macrophage differentiation	7	1	0.11	0.02445

Biological Process	GO:2001237	negative regulation of extrinsic apoptotic signaling pathway	13	1	0.2	0.02482
Biological Process	GO:0046530	photoreceptor cell differentiation	34	1	0.53	0.02623
Biological Process	GO:0048536	spleen development	24	1	0.38	0.02652
Biological Process	GO:0043550	regulation of lipid kinase activity	22	1	0.34	0.02655
Biological Process	GO:0010256	endomembrane system organization	148	1	2.32	0.02699
Biological Process	GO:0046777	protein autophosphorylation	88	2	1.38	0.02706
Biological Process	GO:0000070	mitotic sister chromatid segregation	42	1	0.66	0.02714
Biological Process	GO:0042551	neuron maturation	17	2	0.27	0.02719
Biological Process	GO:0045685	regulation of glial cell differentiation	31	1	0.49	0.02746
Biological Process	GO:0046835	carbohydrate phosphorylation	7	1	0.11	0.02903
Biological Process	GO:0030182	neuron differentiation	465	10	7.28	0.03003
Biological Process	GO:0071466	cellular response to xenobiotic stimulus	14	1	0.22	0.03032
Biological Process	GO:0048662	negative regulation of smooth muscle cell proliferation	10	1	0.16	0.03106
Biological Process	GO:0035023	regulation of Rho protein signal transduction	85	1	1.33	0.03179
Biological Process	GO:0050869	negative regulation of B cell activation	12	1	0.19	0.03238
Biological Process	GO:0006470	protein dephosphorylation	82	1	1.28	0.03302
Biological Process	GO:0034097	response to cytokine	168	3	2.63	0.0343
Biological Process	GO:0003333	amino acid transmembrane transport	10	2	0.16	0.03477
Biological Process	GO:0001933	negative regulation of protein phosphorylation	104	2	1.63	0.03608
Biological Process	GO:0043412	macromolecule modification	1457	20	22.8	0.03661
Biological Process	GO:0051336	regulation of hydrolase activity	351	5	5.49	0.03724
Biological Process	GO:0002449	lymphocyte mediated immunity	85	3	1.33	0.03773
Biological Process	GO:0006464	cellular protein modification process	1397	20	21.86	0.03862
Biological Process	GO:0036211	protein modification process	1397	20	21.86	0.03862
Biological Process	GO:0031344	regulation of cell projection organization	134	2	2.1	0.04027
Biological Process	GO:0055081	anion homeostasis	17	2	0.27	0.04081
Biological Process	GO:0030853	negative regulation of granulocyte differentiation	6	1	0.09	0.04155
Biological Process	GO:0032729	positive regulation of interferon-gamma production	14	1	0.22	0.04171
Biological Process	GO:1903362	regulation of cellular protein catabolic process	56	1	0.88	0.04187
Biological Process	GO:0006096	glycolytic process	32	2	0.5	0.04194
Biological Process	GO:0006801	superoxide metabolic process	24	1	0.38	0.04202
Biological Process	GO:0009894	regulation of catabolic process	323	4	5.05	0.04276
Biological Process	GO:0007351	tripartite regional subdivision	7	1	0.11	0.04285
Biological Process	GO:0008595	anterior/posterior axis specification, embryo	7	1	0.11	0.04285
Biological Process	GO:0007346	regulation of mitotic cell cycle	148	3	2.32	0.0436
Biological Process	GO:0033124	obsolete regulation of GTP catabolic process	149	2	2.33	0.04382
Biological Process	GO:0001754	eye photoreceptor cell differentiation	28	1	0.44	0.04476
Biological Process	GO:0031329	regulation of cellular catabolic process	287	4	4.49	0.04557
Biological Process	GO:0032446	protein modification by small protein conjugation	269	2	4.21	0.04667
Biological Process	GO:0006090	pyruvate metabolic process	43	2	0.67	0.04674
Biological Process	GO:0010769	regulation of cell morphogenesis involved in differentiation	90	2	1.41	0.04786
Biological Process	GO:0072595	maintenance of protein localization in organelle	10	1	0.16	0.04922
Biological Process	GO:0001816	cytokine production	179	3	2.8	0.04994
Cellular Component	GO:0070062	extracellular exosome	96	4	1.64	1.00E-30

Cellular Component	GO:0042612	MHC class I protein complex	35	2	0.6	5.30E-11
Cellular Component	GO:0016021	integral component of membrane	2181	56	37.31	7.9E-08
Cellular Component	GO:0005882	intermediate filament	213	2	3.64	8.2E-08
Cellular Component	GO:0005654	nucleoplasm	480	5	8.21	1.1E-07
Cellular Component	GO:0016020	membrane	3787	91	64.78	9.4E-06
Cellular Component	GO:0035189	Rb-E2F complex	7	1	0.12	0.00024
Cellular Component	GO:0045111	intermediate filament cytoskeleton	239	2	4.09	0.00033
Cellular Component	GO:0005819	spindle	124	2	2.12	0.00063
Cellular Component	GO:0005739	mitochondrion	839	14	14.35	0.00187
Cellular Component	GO:0005737	cytoplasm	4158	64	71.12	0.00643
Cellular Component	GO:0031301	integral component of organelle membrane	115	4	1.97	0.00821
Cellular Component	GO:0042383	sarcolemma	37	1	0.63	0.00938
Cellular Component	GO:0044444	cytoplasmic part	2817	45	48.19	0.01005
Cellular Component	GO:0005903	brush border	12	2	0.21	0.01313
Cellular Component	GO:0005768	endosome	230	8	3.93	0.01474
Cellular Component	GO:0012505	endomembrane system	1245	27	21.3	0.01736
Cellular Component	GO:0098552	side of membrane	107	2	1.83	0.01977
Cellular Component	GO:0044432	endoplasmic reticulum part	249	4	4.26	0.02283
Cellular Component	GO:0009897	external side of plasma membrane	84	2	1.44	0.02583
Cellular Component	GO:0031090	organelle membrane	866	20	14.81	0.02809
Cellular Component	GO:0044425	membrane part	2712	66	46.39	0.03034
Cellular Component	GO:0098588	bounding membrane of organelle	590	17	10.09	0.03101
Cellular Component	GO:0031901	early endosome membrane	20	2	0.34	0.0317
Cellular Component	GO:0051233	spindle midzone	8	1	0.14	0.03312
Cellular Component	GO:0043034	costamere	7	1	0.12	0.03946
Molecular Function	GO:0003964	RNA-directed DNA polymerase activity	74	1	1.26	1.00E-30
Molecular Function	GO:0044822	RNA binding	84	1	1.44	4.80E-18
Molecular Function	GO:0042605	peptide antigen binding	38	2	0.65	3.40E-12
Molecular Function	GO:0005200	structural constituent of cytoskeleton	205	1	3.5	4.8E-08
Molecular Function	GO:0003723	RNA binding	521	3	8.9	2.3E-07
Molecular Function	GO:0003824	catalytic activity	3923	61	67.02	5.6E-07
Molecular Function	GO:0003823	antigen binding	44	2	0.75	0.000023
Molecular Function	GO:0004908	interleukin-1 receptor activity	10	1	0.17	0.00045
Molecular Function	GO:0031402	sodium ion binding	6	1	0.1	0.00194
Molecular Function	GO:0031625	ubiquitin protein ligase binding	97	3	1.66	0.00256
Molecular Function	GO:0017022	myosin binding	13	1	0.22	0.00269
Molecular Function	GO:0042802	identical protein binding	444	8	7.59	0.00446
Molecular Function	GO:0005242	inward rectifier potassium channel activity	11	2	0.19	0.00519
Molecular Function	GO:0004497	monooxygenase activity	73	1	1.25	0.00526
Molecular Function	GO:0016705	oxidoreductase activity, acting on paired donors, with incorporation or reduction of molecular oxygen	102	1	1.74	0.00629
Molecular Function	GO:0005088	Ras guanyl-nucleotide exchange factor activity	49	1	0.84	0.00742
Molecular Function	GO:0019899	enzyme binding	535	6	9.14	0.00797
Molecular Function	GO:0051219	phosphoprotein binding	32	3	0.55	0.00841
Molecular Function	GO:0005524	ATP binding	993	18	16.96	0.00917

Molecular Function	GO:0005506	iron ion binding	114	3	1.95	0.01124
Molecular Function	GO:0002162	dystroglycan binding	7	1	0.12	0.01139
Molecular Function	GO:0004675	transmembrane receptor protein serine/threonine kinase activity	11	1	0.19	0.01333
Molecular Function	GO:0005024	transforming growth factor beta-activated receptor activity	11	1	0.19	0.01333
Molecular Function	GO:0043169	cation binding	2134	37	36.46	0.01345
Molecular Function	GO:0016247	channel regulator activity	28	1	0.48	0.01359
Molecular Function	GO:0005520	insulin-like growth factor binding	19	3	0.32	0.01527
Molecular Function	GO:0016787	hydrolase activity	1592	28	27.2	0.01593
Molecular Function	GO:0046872	metal ion binding	2096	37	35.81	0.01637
Molecular Function	GO:0042803	protein homodimerization activity	279	3	4.77	0.01677
Molecular Function	GO:0001102	RNA polymerase II activating transcription factor binding	31	1	0.53	0.01761
Molecular Function	GO:0005057	signal transducer activity, downstream of receptor	44	2	0.75	0.01866
Molecular Function	GO:0001085	RNA polymerase II transcription factor binding	53	1	0.91	0.02003
Molecular Function	GO:0016820	hydrolase activity, acting on acid anhydrides, catalyzing transmembrane movement of substances	81	3	1.38	0.02639
Molecular Function	GO:0008373	sialyltransferase activity	23	1	0.39	0.02685
Molecular Function	GO:0016788	hydrolase activity, acting on ester bonds	475	7	8.11	0.02847
Molecular Function	GO:0004896	cytokine receptor activity	49	3	0.84	0.02984
Molecular Function	GO:0017111	nucleoside-triphosphatase activity	536	10	9.16	0.03028
Molecular Function	GO:0061630	ubiquitin protein ligase activity	11	1	0.19	0.03066
Molecular Function	GO:0003774	motor activity	78	2	1.33	0.03194
Molecular Function	GO:0015293	symporter activity	49	3	0.84	0.03202
Molecular Function	GO:0004175	endopeptidase activity	226	3	3.86	0.03285
Molecular Function	GO:0043492	ATPase activity, coupled to movement of substances	79	3	1.35	0.03347
Molecular Function	GO:0016740	transferase activity	1532	25	26.17	0.03402
Molecular Function	GO:0051117	ATPase binding	13	1	0.22	0.0349
Molecular Function	GO:0016772	transferase activity, transferring phosphorus-containing groups	759	13	12.97	0.03915
Molecular Function	GO:0008324	cation transmembrane transporter activity	369	14	6.3	0.03935
Molecular Function	GO:0042626	ATPase activity, coupled to transmembrane movement of substances	77	3	1.32	0.0423
Molecular Function	GO:0020037	heme binding	95	3	1.62	0.0424
Molecular Function	GO:0017171	serine hydrolase activity	104	1	1.78	0.04303
Molecular Function	GO:0005509	calcium ion binding	453	3	7.74	0.0437
Molecular Function	GO:0046914	transition metal ion binding	914	16	15.61	0.04378
Molecular Function	GO:0046906	tetrapyrrole binding	101	3	1.73	0.04552
Molecular Function	GO:0043167	ion binding	3602	61	61.54	0.04654
Molecular Function	GO:0015296	anion:cation symporter activity	19	1	0.32	0.04679
Molecular Function	GO:0015399	primary active transmembrane transporter activity	81	3	1.38	0.04699
Molecular Function	GO:0015405	P-P-bond-hydrolysis-driven transmembrane transporter activity	81	3	1.38	0.04699
Molecular Function	GO:0004867	serine-type endopeptidase inhibitor activity	40	1	0.68	0.04727
Molecular Function	GO:0016818	hydrolase activity, acting on acid anhydrides, in phosphorus-containing anhydrides	563	10	9.62	0.04774
Molecular Function	GO:0008236	serine-type peptidase activity	103	1	1.76	0.04786
Molecular Function	GO:0001047	core promoter binding	40	1	0.68	0.04815
Molecular Function	GO:0016462	pyrophosphatase activity	559	10	9.55	0.04852

Molecular Function	GO:0005048	signal sequence binding	10	1	0.17	0.04913
Molecular Function	GO:0004252	serine-type endopeptidase activity	84	1	1.44	0.04922

Supplementary Figures

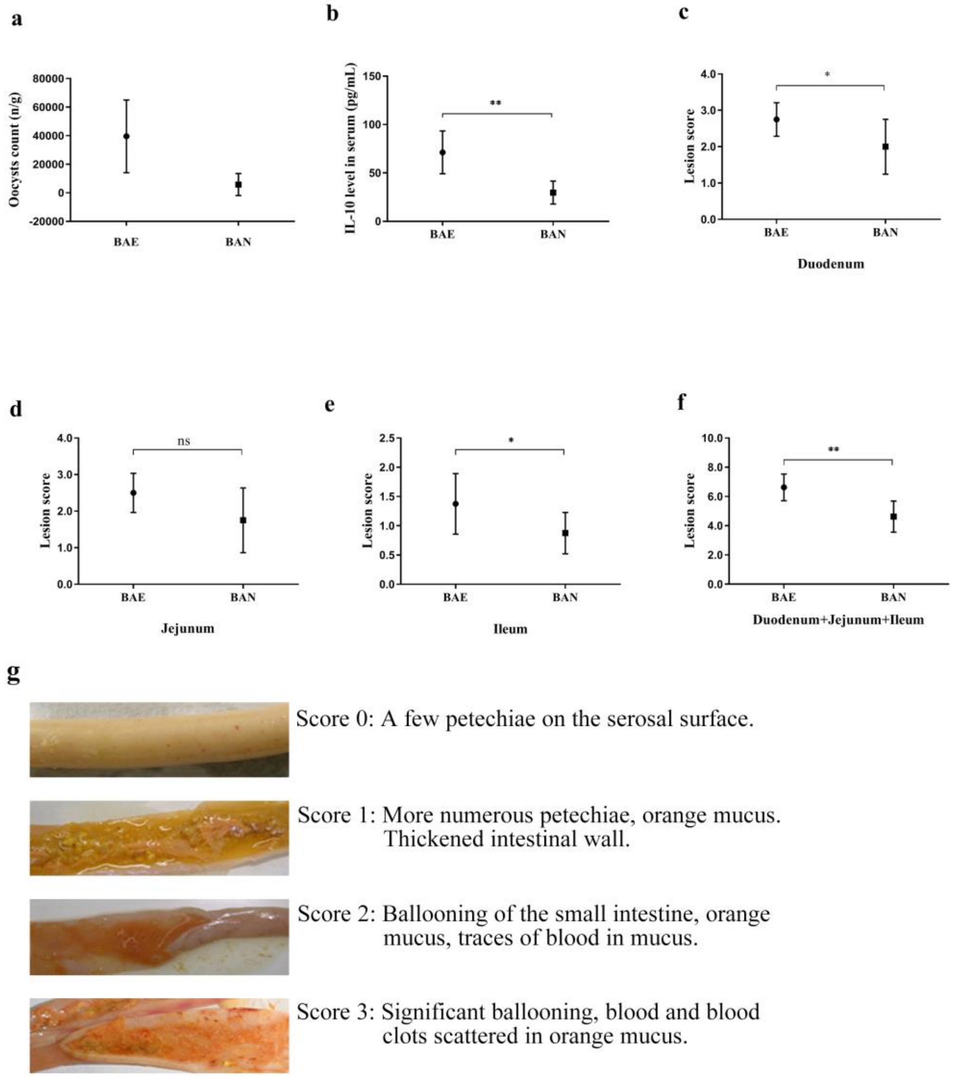

Supplementary Figure 1. The lesion phenotypic and lesion score standard. (a) The oocysts count in feces from BAE group and BAN group. (b) Levels of IL-10 in serum. (c) The lesion score in duodenum. (d) The lesion score in jejunum. (e) The lesion score in ileum. (f) Integral intestinal lesion score. (g) Intestinal lesion score standard used in this study. The data was shown as mean ± SEM; *: $P < 0.05$, **: $P < 0.01$, ns: no significant difference.

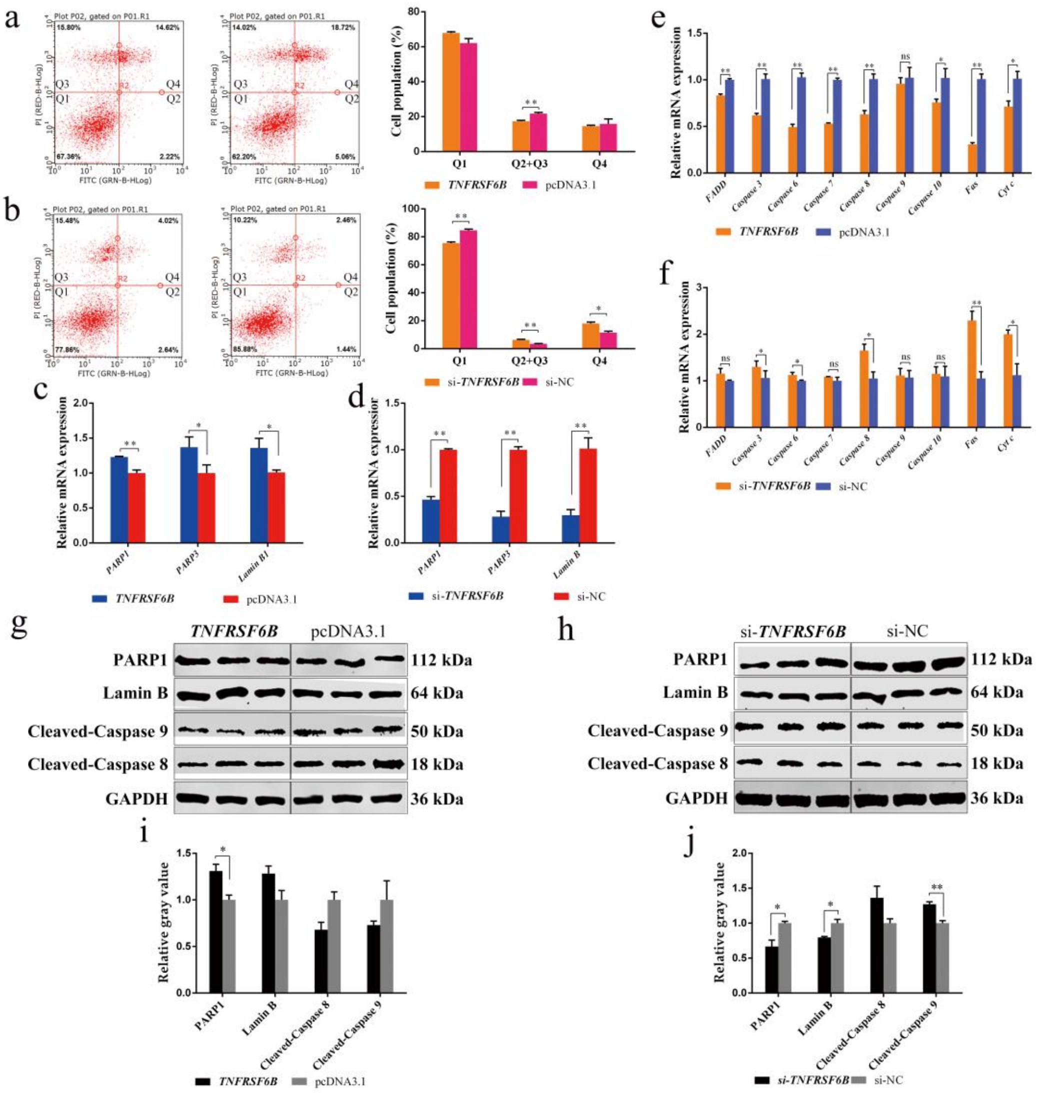

Supplementary Figure 2. *TNFRSF6B* inhibits macrophages apoptosis by downregulating Fas signal pathway. (a) Flow cytometry of Annexin V-FITC and propidium iodide (PI) dual staining measuring the apoptosis of HD11 cells after transfection of pcDNA3.1-*TNFRSF6B*. (b) Flow cytometry of Annexin V-FITC and propidium iodide (PI) dual staining measuring the apoptosis of HD11 cells after transfection of si-*TNFRSF6B*. (c) The mRNA expression of anti-apoptosis genes was upregulated after overexpression of *TNFRSF6B* by qPCR. (d) The mRNA expression of anti-apoptosis genes was downregulated after knockdown of *TNFRSF6B* by qPCR. (e) The mRNA expression of Fas pathway-related genes was downregulated after overexpression of *TNFRSF6B* by qPCR. (f)The mRNA expression of Fas pathway-related genes was upregulated after knockdown of *TNFRSF6B* by qPCR. (g) The protein levels of PARP1, Lamin B, Cleaved-Caspase 8 and Cleaved-Caspase 9 were respectively upregulated and downregulated after overexpression of *TNFRSF6B*. (h) The protein levels of PARP1, Lamin B, Cleaved-Caspase 8 and Cleaved-Caspase 9 were respectively upregulated and downregulated after knockdown of *TNFRSF6B*. (i) The gray value analysis of protein bands in (g). (j) The gray value analysis of protein bands in (h). The data was shown as mean ± SEM; *: P < 0.05, **: P <0.01, ns: no significant difference.
